# Human Urine Stem Cells Alleviate Pulmonary Fibrosis via Inhibiting Macrophage‐Myofibroblast Transition

**DOI:** 10.1002/advs.76150

**Published:** 2026-06-16

**Authors:** Zhou‐Hang Zhang, Guan‐Lin Guo, Xiao‐Hui Guan, Min Hu, Qi‐Ming Huang, Ding‐Wen Guo, Hao‐Cheng Gu, You‐Qiong Zhuo, Ning Li, Hong‐Bo Xin, Ke‐Yu Deng

**Affiliations:** ^1^ The National Engineering Research Center for Bioengineering Drugs and the Technologies Institute of Translational Medicine Jiangxi Medical College Nanchang University Nanchang P. R. China; ^2^ College of Pharmacy Jiangxi Medical College Nanchang University Nanchang P. R. China; ^3^ Guangzhou Key Laboratory of Forensic Multi‐Omics for Precision Identification School of Forensic Medicine Southern Medical University Guangzhou Guangdong P.R. China; ^4^ College of Life Science Nanchang University Nanchang P. R. China; ^5^ School of Food Science and Technology Nanchang University Nanchang P. R. China

**Keywords:** dickkopf‐1 (DKK1), human urine‐derived stem cells (hUSCs), Idiopathic pulmonary fibrosis(IPF), macrophage‐myofibroblast transition(MMT), Wnt/β‐catenin pathway

## Abstract

Human Idiopathic Pulmonary Fibrosis (IPF) is a progressive and fatal lung disease with unknown etiology and lacking efficient treatments. Here, we reported that human urine stem cells (hUSCs) significantly alleviated pulmonary fibrosis via inhibiting macrophage‐myofibroblast transition (MMT), which was identified as a pivotal pathological process in IPF, with the strong interaction among infiltrated macrophages, damaged alveolar epithelial cells, and myofibroblasts via single‐nucleus RNA sequencing data analysis and co‐immunostaining. In addition, hUSCs significantly alleviated pulmonary fibrosis by attenuating alveolar epithelial cell damage, reducing monocyte‐derived macrophage infiltration, and suppressing MMT in the bleomycin‐induced pulmonary fibrosis mouse model. Furthermore, we demonstrated hUSCs inhibited monocyte recruitment and MMT via paracrine actions in the macrophage‐alveolar epithelial cell co‐culture system. Mechanistically, DKK1, which was highly secreted by hUSCs and identified by Venn diagram analysis between the luminex assay in supernatants of THP1 treated with hUSC‐CM and antibody array of hUSC‐CM, might contribute to preventing MMT via suppressing the Wnt/β‐catenin signaling pathway in macrophages. In summary, hUSCs exerted multifaceted protective effects against pulmonary fibrosis, at least in part through paracrine mechanisms involving DKK1 and its modulation of Wnt/β‐catenin‐associated fibrotic responses in MMT. Therefore, hUSCs might provide a potential therapeutic strategy for IPF clinically.

## Graphical abstract

Therapeutic role and mechanism of human urine stem cells (hUSCs) in pulmonary fibrosis. hUSCs alleviated pulmonary fibrosis by selectively inhibiting macrophage‐myofibroblast transition (MMT) in two ways: on one hand, hUSCs inhibited mitochondrial reactive oxygen species (mtROS) production and apoptosis/senescence of epithelial cells in pulmonary fibrosis; on the other hand, hUSC‐derived DKK1 suppressed monocyte recruitment and MMT through blocking the Wnt/β‐catenin/TGF‐β pathway in macrophages and ultimately attenuating pulmonary fibrosis.

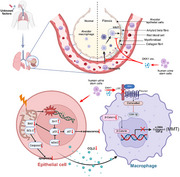

## 1 Introduction

Idiopathic pulmonary fibrosis (IPF) is a progressive and irreversible interstitial lung disease with a low to 48% of 5‐year survival rate, in which its main pathological features are alveolar structural destruction and excessive extracellular matrix deposition [1]. As a critical interface between lung tissue and the external environment, alveolar epithelial cells play a pivotal role in protecting the lung from various pathogens, whereas the disruption of their barrier function is the key initiating factor for the onset and progression of IPF [2, 3, 4, 5]. During the fibrotic process, activated myofibroblasts serve as the primary drivers of excessive collagen deposition. While it has been well established that myofibroblasts originate from the activation of resident lung fibroblasts or from the epithelial‐mesenchymal transition (EMT) of alveolar epithelial cells [6], emerging evidence suggests that macrophage‐myofibroblast transition (MMT) may represent another important source of pulmonary fibrosis [7, 8, 9]. Studies indicated that radiation and/or bleomycin (BLM)‐induced epithelial cells’ damage recruited macrophages via activating the p21‐CCL7 axis, thereby inducing and/or exacerbating pulmonary fibrosis [10]. However, the interaction between inflammatory/immune responses and local tissue injury/fibrosis during IPF progression, as well as the underlying regulatory mechanisms, remains to be elucidated. Particularly, there is a lack of in‐depth clinical‐level analysis [11], which significantly constrains the development and optimization of targeted therapeutic strategies.

Macrophages play a pivotal role in connecting tissue injury and fibrotic repair during IPF progression, and their functional states directly regulate the balance between tissue repair and fibrosis [12]. In the chronic injury microenvironment, macrophages undergo abnormal polarization toward a pro‐fibrotic M2 phenotype [13], in which the macrophages persistently secrete TGF‐β to activate myofibroblasts and disrupt extracellular matrix homeostasis, and even directly participate in fibrotic scar formation through MMT. Numerous studies have confirmed that selective depletion or inhibition of macrophages significantly alleviated experimental pulmonary fibrosis [14], establishing macrophages, particularly those with a pathological M2 phenotype, as a potential key therapeutic target for pulmonary fibrosis treatment.

Despite the identification of macrophages as a potential key therapeutic target, current approaches for treatment of IPF were limited due to the lack of effective antifibrotic medications [15]. Notably, the antifibrotic drugs such as pirfenidone and nintedanib are not able to improve the overall mortality of IPF patients due to their presenting numerous adverse effects [16, 17]. Therefore, exploring safe and effective treatments for IPF is urgently needed, which holds significant clinical importance for ultimately improving lung function and prolonging the survival of patients with IPF. Cell‐based therapy, especially the therapy based on mesenchymal stem cells (MSCs), offers a novel approach for treating fibrotic diseases [18, 19, 20]. However, the clinical translation of conventional MSCs is constrained by issues such as limited sources, immunogenicity, and tumorigenic risks [21]. Human urine stem cells (hUSCs) exhibit unique advantages due to their non‐invasive acquisition, low immunogenicity, high proliferative capacity, and multipotent differentiation potential [22]. Nevertheless, the role of hUSCs in pulmonary fibrosis is still not clear.

In the present study, we have established a serum‐free culture system to obtain high‐purity hUSCs, in which their therapeutic effects in a bleomycin‐induced mouse model of pulmonary fibrosis were evaluated. Focusing on the “alveolar epithelial cell–macrophage–myofibroblast” regulatory axis, we further investigated the therapeutic effects and underlying mechanism of hUSCs on pulmonary fibrosis, and our results demonstrated that hUSCs might provide a promising approach for the treatment of IPF through inhibiting macrophage‐myofibroblast transition (MMT) in pulmonary fibrosis clinically.

## 2 Results

### 2.1 Identification and Characteristics of hUSCs

**FIGURE 1:**
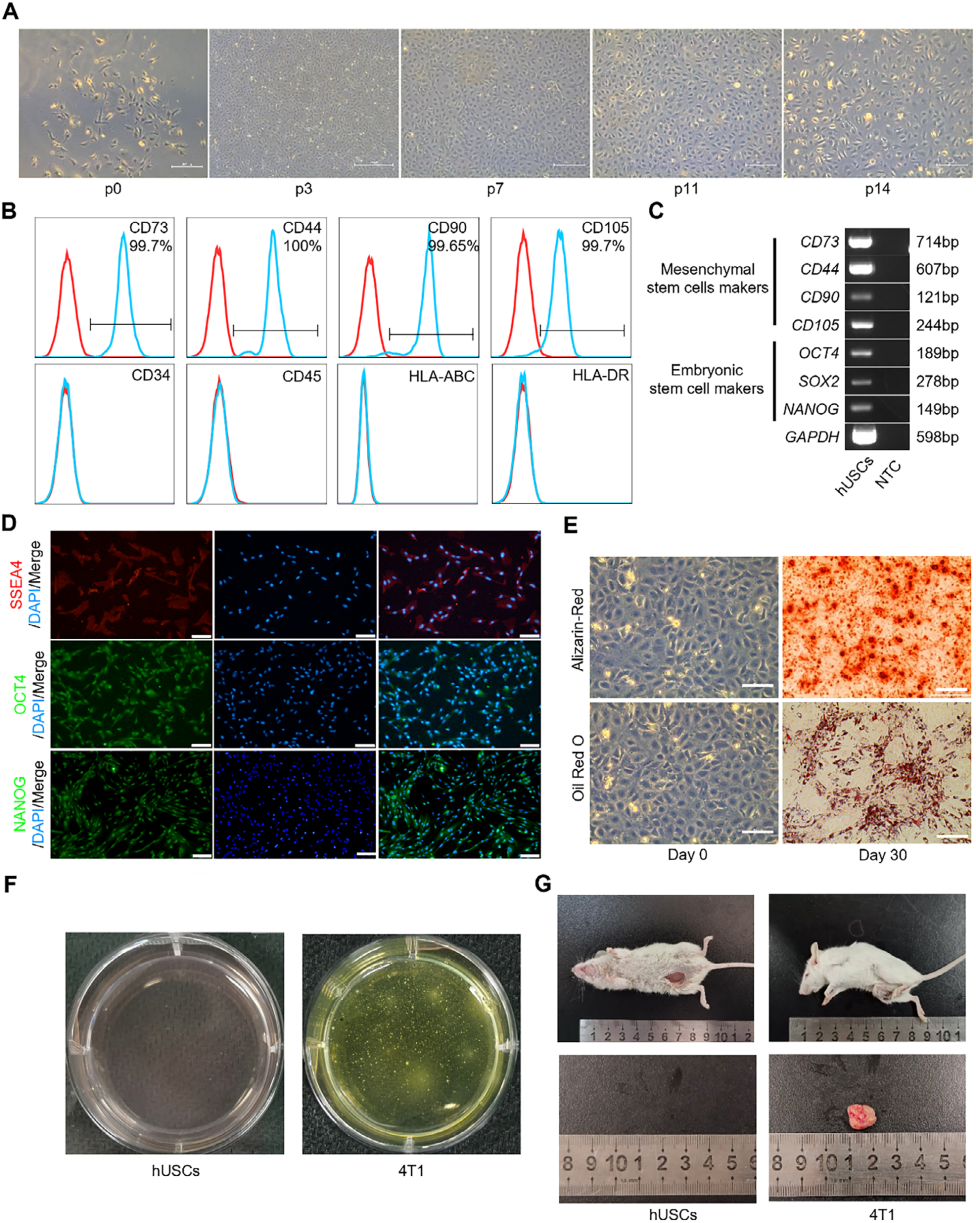
Identifications and characteristics of human urine‐derived stem cells (hUSCs). (A) The morphological photographs of human urine‐derived stem cells (hUSCs) were taken by inverted microscope at serial passages (Scale bar = 200 µm) in a serum‐free culture condition. (B) The expressions of the cell surface markers such as CD73, CD44, CD90, CD105, CD34, CD45, HLA‐ABC and HLA‐DR of hUSCs were examined by flow cytometry. (C) Agarose gel electrophoresis of RT‐PCR products showing the mRNA expression of *CD73, CD44, CD90, CD105, OCT4, SOX2* and *NANOG* in hUSCs. Water was used as the no‐template control (NTC). (D) Representative images of immunofluorescent staining of the stem cell surface markers Oct4, SSEA‐4, and Nanog taken in hUSCs. (E) The multi‐differentiational potentials of hUSCs were determined by osteogenesis with alizarin red staining, and adipogenesis with oil red O staining, respectively. (F) The tumorigenicities of hUSCs and 4T1 cells were assessed in soft agar culture in vitro. (G) The tumorigenicities of hUSCs or 4T1 tumor cells were determined in NOD‐SCID mice by subcutaneous injection of 1 × 10^6^ cells in 100 µL PBS, respectively.

Human urine stem cells (hUSCs) with a cobblestone‐like shape appeared within 5–7 days in an optimized serum‐free culture medium (SF medium). Notably, hUSCs maintained a high and stable self‐renewal status potentially for up to 14 passages in SF medium (Figure 1A). Flow cytometry analysis demonstrated that hUSCs expressed the mesenchymal surface markers CD73, CD90, CD105, and CD44, but were negative for the hematopoietic markers CD34 and CD45 as well as the major histocompatibility complex molecules HLA‐ABC and HLA‐DR (Figure 1B). In parallel, RT‐PCR analysis confirmed the expression of mesenchymal marker genes (*CD73*, *CD44*, *CD90*, and *CD105*) and pluripotency‐associated genes (*OCT4*, *SOX2*, and *NANOG*) (Figure 1C). In addition, the expressions of SSEA4, OCT4, and NANOG were confirmed by immunofluorescence staining (Figure 1D). Furthermore, the multipotent potentials of hUSCs were demonstrated with adipogenesis and osteogenesis, as indicated by positive Oil Red O and Alizarin Red staining, respectively (Figure 1E).

Soft agar assay (in vitro) and NOD/SCID immuno‐deficient mice (in vivo) were used to assess the tumorigenesis of hUSCs and 4T1 cells (mouse breast cancer cells), respectively, and the results showed that hUSCs did not form the colonies or tumors during the observation period (up to 30 days in vitro and 20 weeks in vivo), whereas 4T1 cells formed obvious colonies in soft agar within 30 days of culture and tumors in NOD/SCID mice within 7–9 weeks (Figure 1F, G). Collectively, these results defined that hUSCs possessed a potent self‐renewal capacity, multilineage differentiation potential, and low/no immunogenicity.

### 2.2 hUSCs Attenuate BLM‐Induced Pulmonary Fibrosis in Mice

**FIGURE 2:**
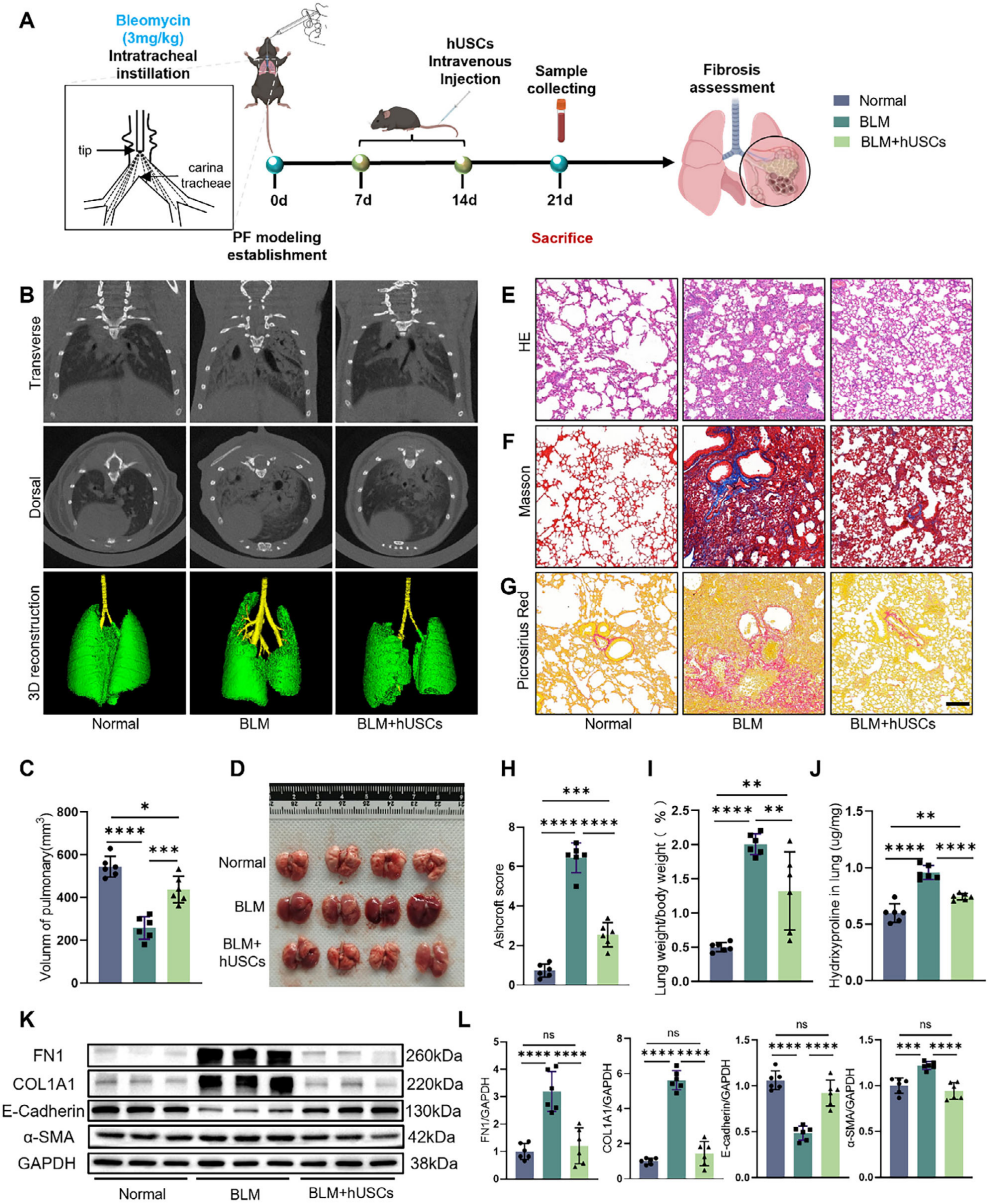
hUSCs ameliorate mouse pulmonary fibrosis induced by Bleomycin (BLM). (A) Illustration of BLM‐induced mouse pulmonary fibrosis (BLM‐PF) models and the administration of hUSCs in vivo. (B) Representative coronal 2D micro‐ computed tomography (CT) lung slices (top) and their corresponding 3D renderings (below) were obtained by lung CT analysis in mice with various treatments. (C) Statistical analysis of lung volume from Figure 2B. (D) Representative mouse lung photos were taken at the end of experiments. (E–G) Representative images of H&E, Masson and Sirius Red staining were taken in mouse lung tissues (Scale bar = 200 µm). (H) Ashcroft scores were obtained based on pathological staining results of mouse lung tissues in each group. (I) The ratio of lung weight to body weight. (J) Hydroxyproline contents in lung tissues. (K, L) Representative images (K) and quantitative results (L) of the expressions of FN1, COL1A1, E‐cadherin, α‐SMA were determined by Western blot in lung tissues. GAPDH was used as loading control. Data were represented as mean ± SD. Significance was measured using one‐way ANOVA. ^*^ *P* < 0.05, ^**^ *P* < 0.01, ^***^ *P* < 0.001, ^****^ *P*<0.0001, n = 6.

As a first‐line antineoplastic agent, bleomycin (BLM) has also been used to induce pulmonary fibrosis (PF) in mice due to its specific cytotoxic properties in the lung, in which it intercalates into DNA and generates reactive oxygen species via iron‐mediated catalysis, causing single‐ or double‐strand DNA breaks to trigger persistent lung tissue damage and aberrant repair [23]. To evaluate the therapeutic potential of hUSCs in pulmonary fibrosis, the cells were intravenously administered into the mice with 1 × 10^6^ cells/mouse on day 7th and 14th post‐BLM (Figure 2A), respectively. Lung computed tomography (CT) analysis showed that the traction bronchiectasis and honeycombing were significantly increased in lung tissues in BLM‐treated mice compared with normal mouse lung tissues, whereas hUSCs markedly ameliorated BLM‐induced lung damage in mice (Figure 2B–D). Immunofluorescence staining with anti‐human nuclei antibody was performed in mouse lung tissues at the end of the experiment, supporting the pulmonary localization/retention of transplanted hUSCs in vivo (Figure S1A). In addition, the results from HE, Masson's trichrome, and Sirius Red staining showed that hUSCs markedly alleviated BLM‐induced alveolar destruction, inflammatory cell infiltration, and septal thickening (Figure 2E–G). Furthermore, hUSCs intervention remarkably reduced BLM‐induced fibrosis (Figure 2H) and collagen deposition (Figure S1B, C), and increment of lung weight to body weight ratios (Figure 2I) and hydroxyproline content (Figure 2J), suggesting that hUSCs might improve BLM‐induced fibrosis and pulmonary edema. Moreover, hUSCs prevented BLM‐induced elevations of myofibroblast markers’ expressions, such as fibronectin (FN1), collagen I (COL1A1), and α‐smooth muscle actin (α‐SMA), and enhanced E‐cadherin expression in lung tissues in BLM‐treated mice (Figure 2K, L). Consistently, RT‐qPCR analysis showed that the mRNA expression levels of *Fn1*, *Col1a1*, and *Acta2* were also significantly increased in the BLM group and were markedly reduced after hUSC treatment (Figure S1D). Taken together, these results demonstrated that hUSCs attenuated BLM‐induced pulmonary fibrosis in mice.

In addition, considering that hUSCs were administered intravenously in this therapeutic model, we further evaluated their in vivo biosafety after systemic delivery. Histological examination of major organs such as hearts, liver, spleen, and kidney revealed no obvious pathological abnormalities or tissue injury after intravenous injection of hUSCs (Figure S1E), supporting the biosafety of hUSC administration in this study.

### 2.3 hUSCs Ameliorate BLM‐Induced Epithelial Cell Damage

**FIGURE 3:**
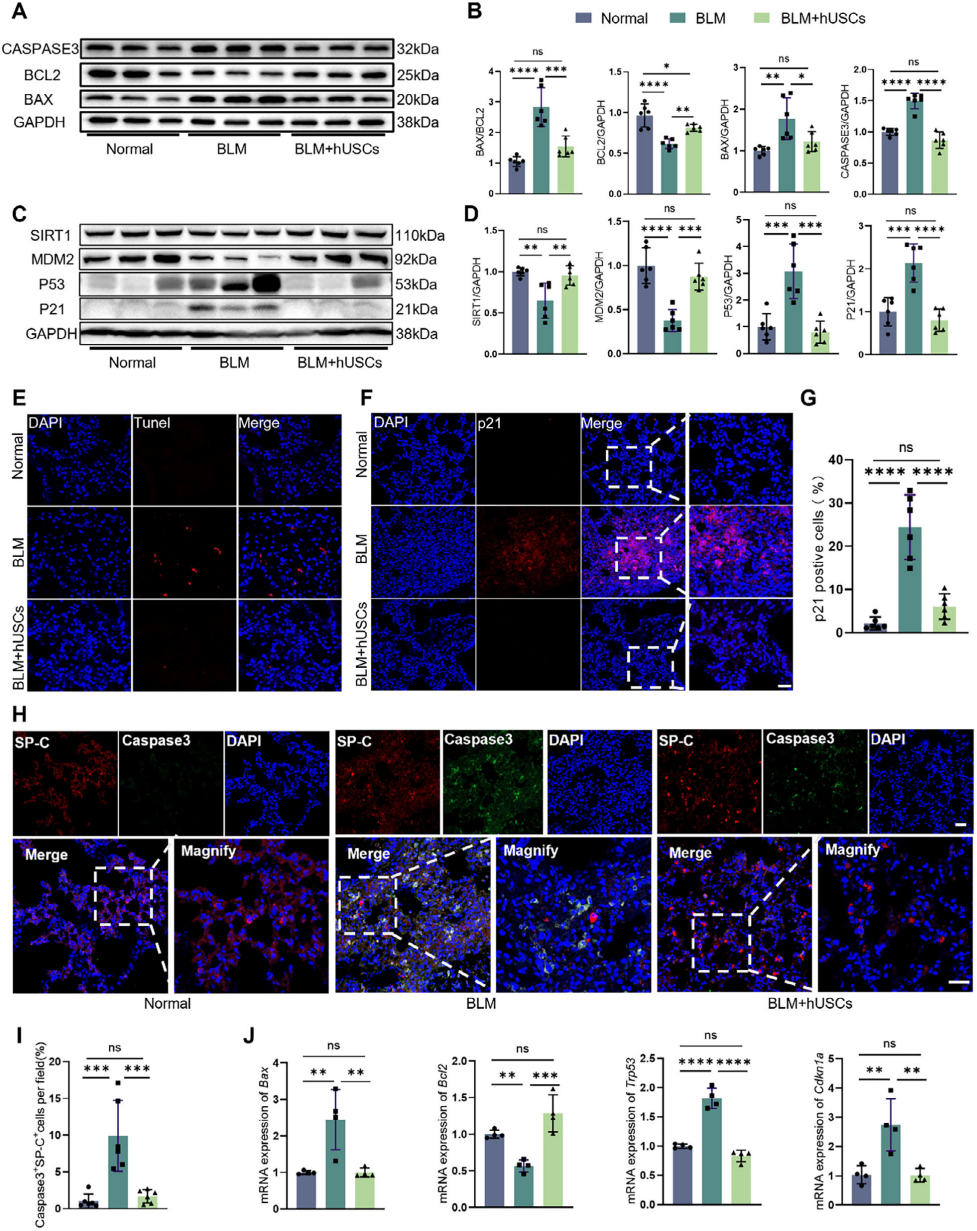
hUSCs attenuate BLM‐induced alveolar epithelial cell damages. (A–D) The representative images (A, C) and quantitative results (B, D) of pulmonary expressions of apoptosis‐related proteins including BAX, BCL‐2 and CASPASE‐3 (A, B) and senescence‐related proteins including SIRT1, MDM2, P53 and P21 (C, D) were determined by western blot. (E) Representative images of TUNEL staining for apoptosis in mouse lung tissues. (F,G) Representative images (F) and quantitative results (G) of p21 expressions in mouse lung tissues, Bar = 20µm. (H) Representative images of immunofluorescence staining of Caspase3 and SP‐C in mouse lung tissues, Bar = 20µm. (I) Quantification of fluorescence intensity in Caspase3^+^/SP‐C^+^ double‐positive cells from H. (J) The relative mRNA expression of *Bax*, *Bcl2*, *Trp53* and *Cdkn1a* were determined by RT‐qPCR in mouse lung tissues, with *Gapdh* as the internal control. Data were represented as mean ± SD. Significance was measured using one‐way ANOVA. ^*^ *P* < 0.05, ^**^ *P* < 0.01, ^***^ *P* < 0.001, ^****^ *P*<0.0001, n = 4–6.

Alveolar epithelial damage and dysfunction, especially in proliferative ATII epithelial cells, have been considered as triggers and consequences in IPF [24]. To further assess the protective effects of hUSCs on BLM‐induced pulmonary damage, cytotoxicity‐related assays were performed. The results revealed that hUSCs significantly reduced BLM‐induced alterations in apoptosis‐related proteins such as BAX, BCL‐2, CASPASE‐3 (Figure 3A, B), and senescence‐related proteins such as SIRT1, MDM2, P53, and P21 (Figure 3C, D) in mouse lung tissues. Our immunofluorescent staining showed that hUSCs markedly suppressed BLM‐induced apoptosis (Figure 3E) and the elevated expression of cellular senescence marker P21 (Figures 3F, G) in lung tissues in BLM‐treated mice. To further identify the main cell type injured by BLM, dual immunofluorescence staining was performed using SP‐C, a marker of type II alveolar epithelial cells, together with the apoptosis marker Caspase‐3. The results showed that hUSCs remarkably alleviated BLM‐induced injury in type II alveolar epithelial cells (Figure 3H, I). Consistently, RT‐qPCR analysis further demonstrated that the mRNA expression levels of *Bax*, *Bcl2*, *Trp53*, and *Cdkn1a* were significantly altered in the BLM group and were partially reversed by hUSC treatment (Figure 3J).

**FIGURE 4:**
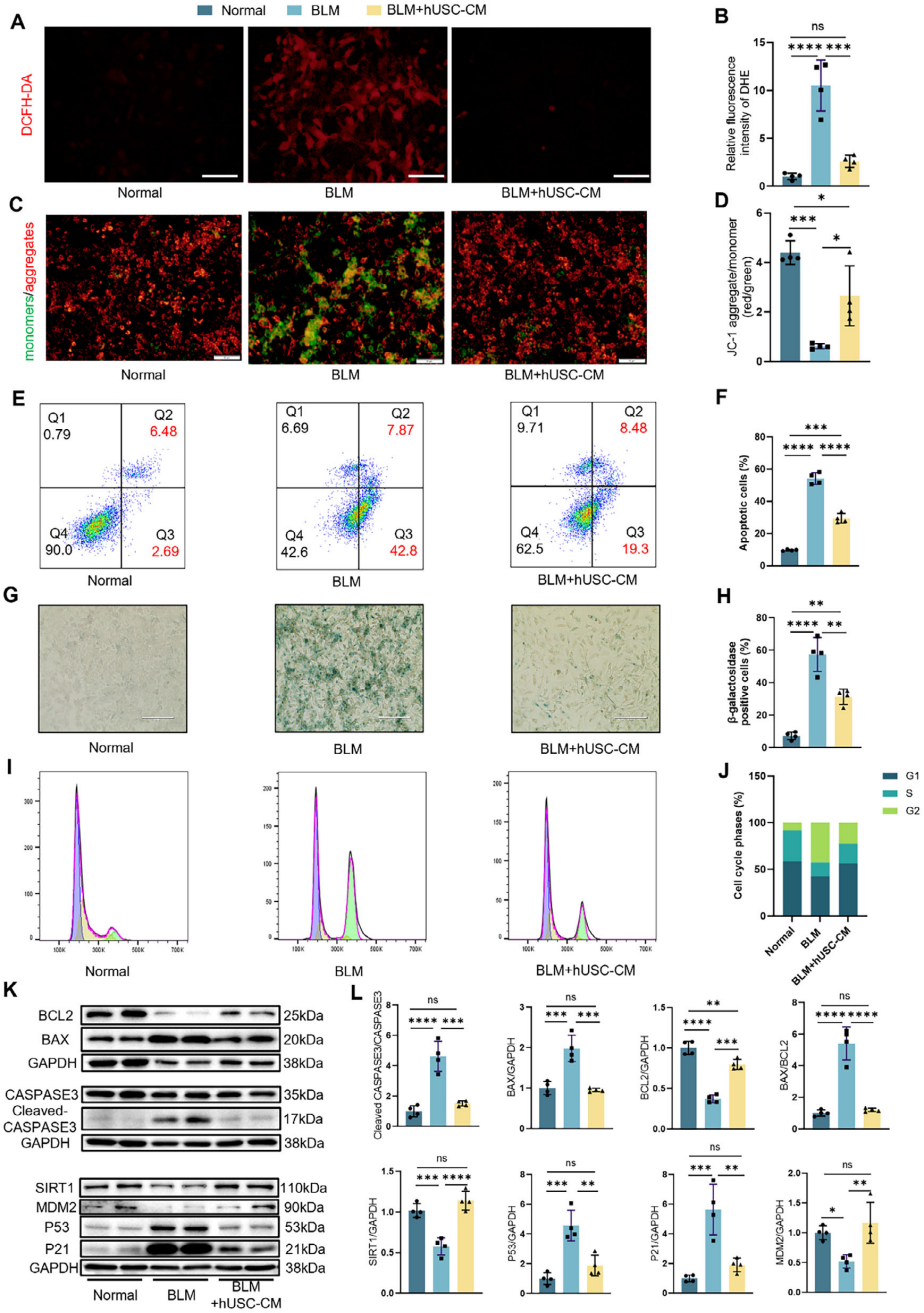
hUSC‐CM ameliorate BLM‐induced impairment of alveolar epithelial cells (A549) in vitro. (A,B) Representative images (A) and quantitative analysis (B) of ROS productions in A549 cells determined by DHE staining (in red) following BLM stimulation. (C,D) Representative images (C) and quantitative analysis of mitochondrial potentials (D) in A549 cells determined by JC‐1 fluorescence Staining. (E,F) Flow charts of flow metry assay (E) and quantitative analysis (F) of apoptosis in A549 cells treated with BLM for 48 h. (G,H) Representative images (G) and quantitative analysis (H) of senescence in A549 cells staining of senescence‐associated β‐galactosidase (SA‐β‐gal) in A549 cells. (I,J) The flow charts of flow cytometry assay (I) and quantitative analysis (J) of cell cycles in A549 cells. (K,L) Representative images (K) and quantitative analysis (L) of the expressions of senescence and apoptosis related proteins in A549 cells. GAPDH was used as loading control. Data were represented as mean ± SD. Significance was measured using a one‐way ANOVA. ^*^ *P* < 0.05, ^**^ *P* < 0.01, ^***^ *P* < 0.001, ^****^ *P*<0.0001, n = 4.

Consistent with the findings in vivo, BLM induced A549 cell death (Figure S2A) and morphological alterations (Figure S2B), and flow cytometric cell cycle analysis showed that BLM caused cell cycle arrest in A549 cells (Figure S2C, D) in a dose‐dependent manner. Western blot analysis showed that BLM upregulated the expressions of pro‐apoptotic (BAX) and senescence‐associated (P53, P21) proteins, and downregulated the expressions of BCL‐2 and Sirt1 in A549 cells (Figure S2E, F). In addition, hUSC conditioned medium (hUSC‐CM) was used to evaluate whether the protective effects of hUSCs were related to their direct or paracrine action. The results revealed that hUSC‐CM significantly suppressed ROS generation (Figure 4A, B), restored mitochondrial membrane potential (Figure 4C, D), and reduced apoptosis (Figure 4E, F and Figure S3A, B) in BLM‐treated A549 cells. In addition, hUSC‐CM alleviated cellular senescence (Figure 4G, H), reversed G2 phase arrest, increased the proportion of cells entering G1 and S phase (Figure 4I, J), and restored the expressions of BAX, BCL‐2, CASPASE‐3, SIRT1, MDM2, P53, and P21 proteins (Figure 4K, L) to eliminate detrimental effects from BLM. Furthermore, hUSC‐CM restored proliferative capacity in A549 cells (Figure S3C, D). Consistently, RT‐qPCR analysis further showed that the mRNA expression levels of *BAX*, *BCL2*, *TP53*, and *CDKN1A* were significantly dysregulated after BLM stimulation and were partially reversed by hUSC‐CM treatment. Notably, the expression of *CCL2* was markedly increased in BLM‐injured alveolar epithelial cells, whereas hUSC‐CM significantly suppressed this increase (Figure S3E). These results indicated that hUSCs protected alveolar epithelial cells against BLM‐induced injury by attenuating oxidative stress and mitochondrial dysfunction, thereby inhibiting apoptosis, senescence, and cell cycle arrest.

### 2.4 Macrophage‐Myofibroblast Transition (MMT) Was Significantly Increased in Lung Tissues with Idiopathic Pulmonary Fibrosis

**FIGURE 5:**
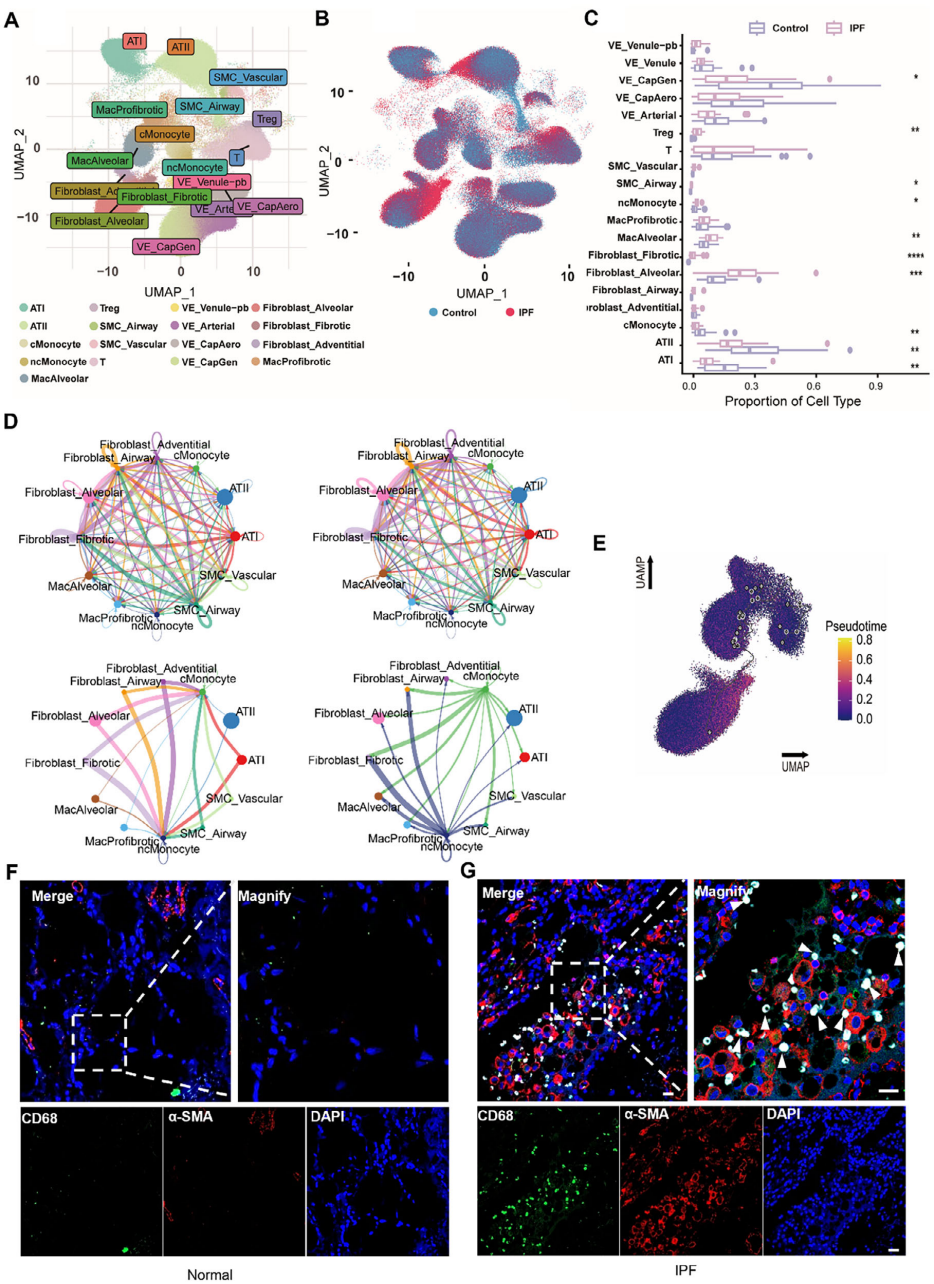
The macrophage‐to‐myofibroblast transition (MMT) is increased in fibrotic lung tissues from IPF patients. (A) The clusters of cells were performed by UMAP dimensionality reduction analysis. (B) UMAP with cells were labeled by disease identity. (C) Proportion distribution of different cell types was analyzed in healthy control and IPF samples. (D) Potential ligand‐receptor crosstalk analysis was performed with monocytes, macrophages, fibroblasts, and epithelial cell subpopulations. (E) The macrophage‐to‐myofibroblast transition was determined by RNA velocity (scVelo) analysis. (F,G) The co‐localization of CD68 and α‐SMA were determined by immunofluorescent staining in human lung tissues, Bar = 20 µm.

To further clarify which cell types may be involved in idiopathic pulmonary fibrosis (IPF), we performed bioinformatics analysis on 65 human lung samples, including 32 IPF patients and 33 healthy donors from the IPF database (GSE286182 dataset). After quality control of the dataset, we conducted UMAP dimensionality reduction and cell annotation, and ultimately, 17 distinct cell types were identified in close association with IPF (Figure 5A). In addition, differential analysis of cell infiltration and subset abundance revealed a significant increase in the abundance of alveolar fibroblasts, alveolar macrophages, and pro‐fibrotic macrophages in fibrotic lung tissues (Figure 5B, C), indicating there were alterations in cell–cell interactions in pulmonary fibrosis. Therefore, we performed cell–cell communication analysis on cell clusters in fibrotic tissues, and the results showed that there were frequent interactions between macrophages, alveolar epithelial cells, and fibroblasts (Figure 5D). To investigate the effects of the interactions, including epithelial cells, monocytes, macrophages, and fibroblasts on IPF, we conducted differential expression analysis of these cell types between idiopathic pulmonary fibrosis (IPF) tissues and normal tissues (Figure S4A), and KEGG and GO enrichment analyses using the identified differentially expressed genes (DEGs). The results showed that both macrophages and fibroblasts in IPF tissues were associated with collagen deposition, extracellular matrix (ECM) signaling pathways, and the IL‐17 signaling pathway (Figure S4B, C), indicating that macrophages might directly participate in pulmonary fibrosis.

In line with the results from KEGG and GO analysis, we found that there were continuously cell state transitions among monocytes, macrophages, and myofibroblasts in fibrotic lung tissues by pseudo time analysis (Figure 5E). Additionally, our immunofluorescence results showed that there were significant co‐localized areas with both macrophage marker CD68 and myofibroblast marker α‐smooth muscle actin (α‐SMA) fibrotic lung tissues from IPF patients (Figure 5F,G). Confocal Z‐stack analysis revealed that the majority of CD68^+^α‐SMA^+^ double‐positive cells were co‐localized within α‐SMA‐positive regions in fibrotic lung tissues (Figure S5 and Video S1). Collectively, these results suggest that MMT may contribute to the development of pulmonary fibrosis. Specifically, initial injury to alveolar epithelial cells may promote monocyte infiltration into lung tissues, followed by macrophage differentiation and subsequent transition toward a myofibroblast‐like phenotype.

### 2.5 hUSCs Inhibit Monocyte Recruitment and Macrophage‐Myofibroblast Transition (MMT) in Pulmonary Fibrosis

**FIGURE 6:**
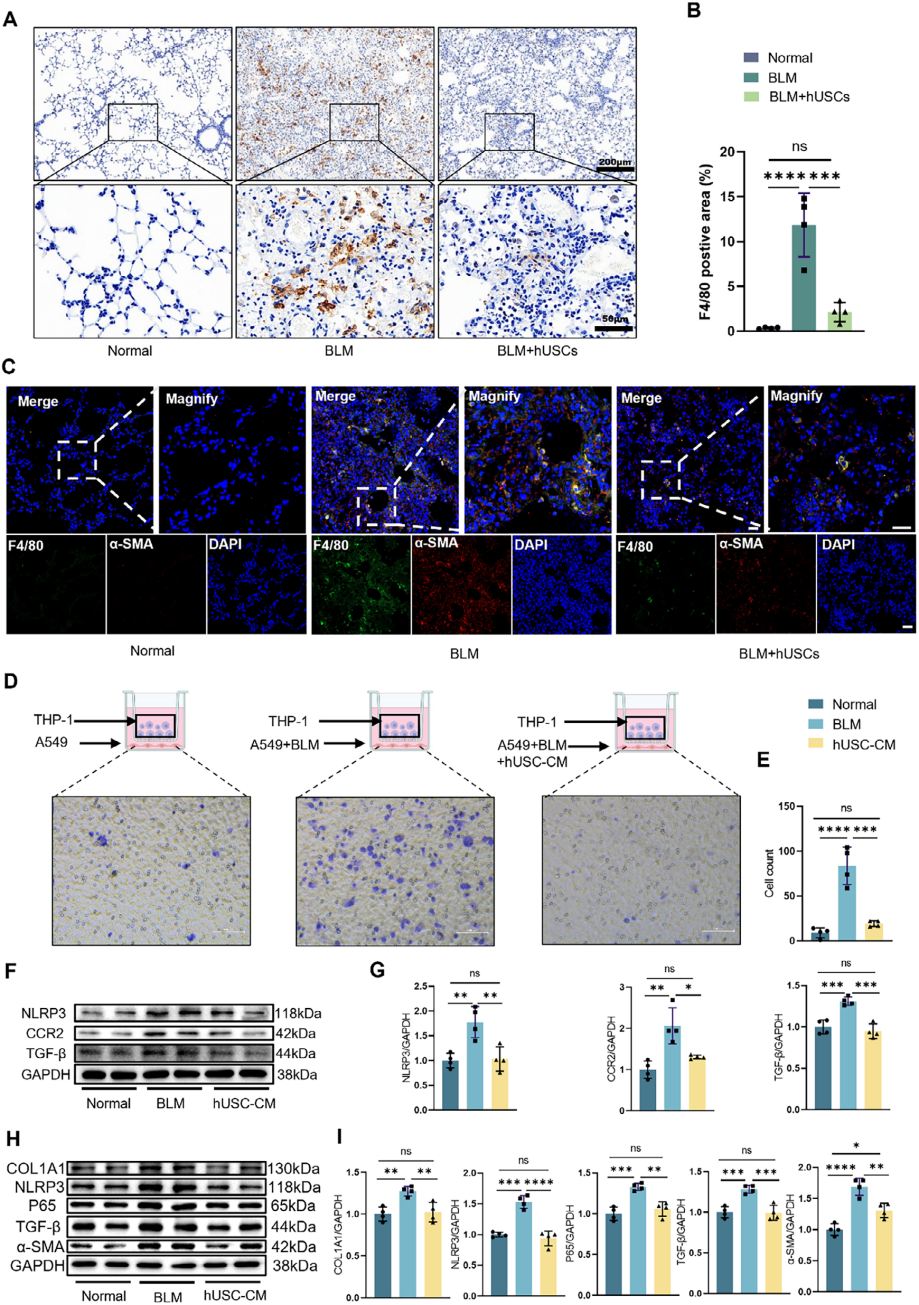
hUSCs attenuated BLM‐induced monocyte recruitment and macrophage‐to‐myofibroblast transition (MMT) in epithelial cells via extracellular components. (A,B) Representative images (A) and quantitative analysis (B) of immunohistochemical staining of F4/80 positive area in mouse lung tissues. (C) The co‐localization of F4/80 and α‐SMA were determined by immunofluorescent staining (bars = 20 µm)in mouse lung tissues. (D,E) The representative images (D) and quantitative analysis (E) of migrated monocyte‐derived macrophages (MDMs) in transwell assay with THP1 and A549 cells. (F,G) Representative images of western blot (F) and quantitative analysis (G) of inflammation‐related proteins in THP‐1 cells. (H,I) Representative images of western blot (H) and quantitative analysis (I) of the expressions of proteins related to macrophage‐to‐myofibroblast transition in THP‐1 cells. Data were represented as mean ± SD. Significance was measured using a one‐way ANOVA. ^*^ *P* < 0.05, ^**^ *P* < 0.01, ^***^ *P* < 0.001, ^****^ *P*<0.0001, n = 4.

ATII epithelial cells are vulnerable to insults from various microinjuries, and their interaction with the immune system has been considered the core link in IPF. Persistent chronic inflammation and immune dysfunction have been gradually recognized to be related to the occurrence and development of IPF, evidenced by the accumulation of a large amount of macrophages in the fibrotic lungs [25]. To assess the role of hUSCs in macrophage infiltration in fibrotic lungs, immunohistochemical staining for the macrophage marker F4/80 and fibrosis marker α‐smooth muscle actin (α‐SMA) was performed. The results showed that hUSCs remarkably attenuated BLM‐induced infiltrations of macrophages (Figure 6A, B) and reduced F4/80^+^α‐SMA^+^ double‐positive cells in fibrotic lung tissues in mice (Figure 6C).

Next, we established a transwell co‐culture system to define the role of hUSCs in the interaction between alveolar epithelial cells and macrophages, and the results showed that hUSC‐CM significantly inhibited the recruitment of monocyte‐derived macrophages, which could be initiated by the danger signals from BLM‐injured epithelial cells (Figure 6D, E). Moreover, BLM‐injured epithelial cells induced the upregulate expression of inflammation‐related proteins (NLRP3, CCR2, TGF‐β) and MMT‐related protein (α‐SMA, COL1A1) in macrophages. These inductions were all remarkably inhibited by hUSC‐CM (Figure 6F–I). In line with the protein‐level findings, RT‐qPCR analysis further demonstrated that the mRNA levels of *COL1A1*, *TGFB1*, *ACTA2*, and *NLRP3* were significantly upregulated in macrophages exposed to supernatants from BLM‐injured epithelial cells, and these changes were markedly reversed by hUSC‐CM treatment (Figure S6). These results indicated that damaged epithelial cells promoted recruitment of monocytes and macrophage‐myofibroblast transition (MMT), whereas the intervention with hUSC‐CM effectively suppressed both processes, suggesting that hUSCs‐mediated protective effects against IPF might be firmly associated with their inhibition of MMT via their paracrine effects.

To further elucidate macrophage‐epithelial crosstalk during injury, we established a multi‐step co‐culture model. First, the culturing medium was collected from alveolar epithelial A549 cells exposed with or without BLM for 48 h. These media were then used to culture THP‐1 monocytes, and the macrophage supernatants (THP1‐SN) were harvested subsequently. Next, Hoechst 33258‐labeled (blue) A549 epithelial cells were stimulated either directly with BLM or indirectly with the macrophage supernatants (A549/BLM‐THP1‐SN) for 48 h and cultured in the presence or absence of hUSCs‐CM for the last 4 h, and then co‐cultured with DiO‐labeled (green) THP‐1 monocytes for an additional 4 h (Figure S7A). In co‐culture assays, THP‐1‐derived macrophages were able to attach to the bottom of the culture dish and robustly phagocytosed the injured A549 cells, and hUSCs significantly reduced the number of attached macrophages and their phagocytosis (Figure S7B–E). Strikingly, the supernatants derived from macrophages (A549/BLM‐THP1‐SN) were far more potent in inducing severe senescent phenotypes in alveolar epithelial cells than direct BLM exposure (Figure S7F). These detrimental indirect effects of BLM between epithelial cells and macrophages (Figure S7G) suggested that paracrine signals from macrophages might amplify epithelial senescence beyond the initial BLM insult.

### 2.6 hUSC‐Derived DDK1 Inhibits MMT via Suppressing Wnt/β‐Catenin Signaling Pathway

**FIGURE 7:**
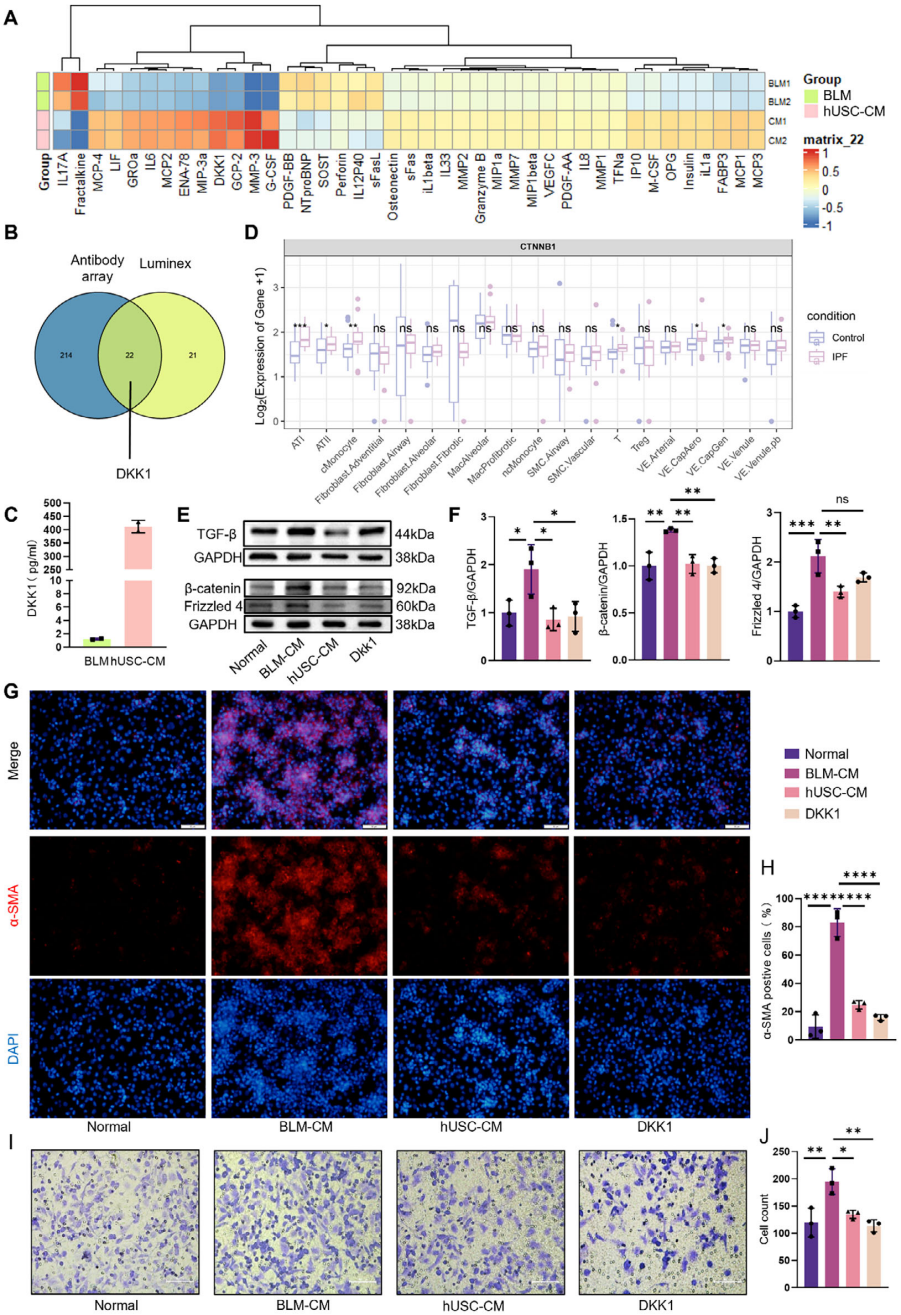
hUSC‐derived DKK1 inhibits BLM‐induced MMT via Wnt/β‐Catenin/ TGF‐β signaling. (A) The cytokine and chemokine profiles were determined by Luminex multiplex assay analysis with the extracellular supernatants from THP‐1 cells treated by BLM or BLM plus hUSC‐CM. (B) Venn diagram of was drawn between upregulated factors of Luminex assay and the results from antibody dot array of hUSC‐CM. (C) The expression levels of DKK1 by Luminex assay as shown in A. (D) Box plots showing the expression of CTNNB1 across cell types in lung tissues from IPF patients and healthy donors. Data are presented as log_2_‐transformed relative expression. (E, F) Representative western blot images (E) and quantitative analysis (F) of proteins related to the Wnt/β‐catenin pathway in THP‐1 macrophages under different treatments. (G) Immunofluorescence staining was performed to detect α‐SMA expression in THP‐1 macrophages under different treatments. (H) Quantitative analysis of immunofluorescence staining of α‐SMA positive area in THP‐1. (I) Macrophage recruitment was assessed by transwell assay with crystal violet staining under co‐culturing condition with A549 cells. (J) Quantitative analysis of migrated monocyte‐derived macrophages (MDMs) in transwell assay. Data were represented as mean ± SD. Significance was measured using a one‐way ANOVA. ^*^ *P* < 0.05, ^**^ *P* < 0.01, ^***^ *P* < 0.001, ^****^ *P*<0.0001, n = 3.

To define the role and mechanism of hUSCs in MMT, we first evaluated and analyzed the secretory profile of monocyte‐derived macrophages (MDMs) following BLM stimulation with or without hUSC‐CM intervention by Luminex assay. A heatmap revealed that hUSCs’ intervention significantly elevated Dickkopf‐1 (DKK1) contents in the secretions (Figure 7A). Venn diagram analysis identified 22 overlapping secreted proteins between hUSC‐CM and the upregulated proteins detected by Luminex assay (Figure 7B). The levels of DKK1 were measured and compared between BLM‐treated macrophages’ supernatants with or without hUSC‐CM (Figure 7C). DKK1 is a well‐known canonical inhibitor of the Wnt/β‐catenin signaling pathway [26, 27]. Furthermore, single‐nucleus RNA sequencing (snRNA‐seq) analysis showed that β‐catenin was highly expressed in alveolar type I (ATI) cells, alveolar type II (ATII) cells, and classical monocytes (cMonocytes) in the fibrotic lung tissues from IPF patients compared with that of healthy donors (Figure 7D). Moreover, both hUSC‐CM and recombinant DKK1 significantly inhibited MMT, which was induced by supernatants from BLM‐injured alveolar epithelial cells. This effect was evidenced by suppressing the expressions of β‐catenin, TGF‐β, Frizzled4 (Figure 7E, F), and α‐SMA (Figure 7G, H) in macrophages, as well as reduced macrophage infiltration (Figure 7I, J). Consistently, RT‐qPCR analysis further demonstrated that the mRNA expression levels of *COL1A1*, *TGFB1*, *ACTA2*, and *AXIN2* were significantly increased in macrophages stimulated with supernatants from BLM‐injured alveolar epithelial cells, whereas hUSC‐CM or recombinant DKK1 markedly attenuated the elevated gene expressions (Figure S8). These data suggested that DKK1, derived from hUSCs, might suppress Wnt‐β‐catenin signaling and inhibit MMT.

**FIGURE 8:**
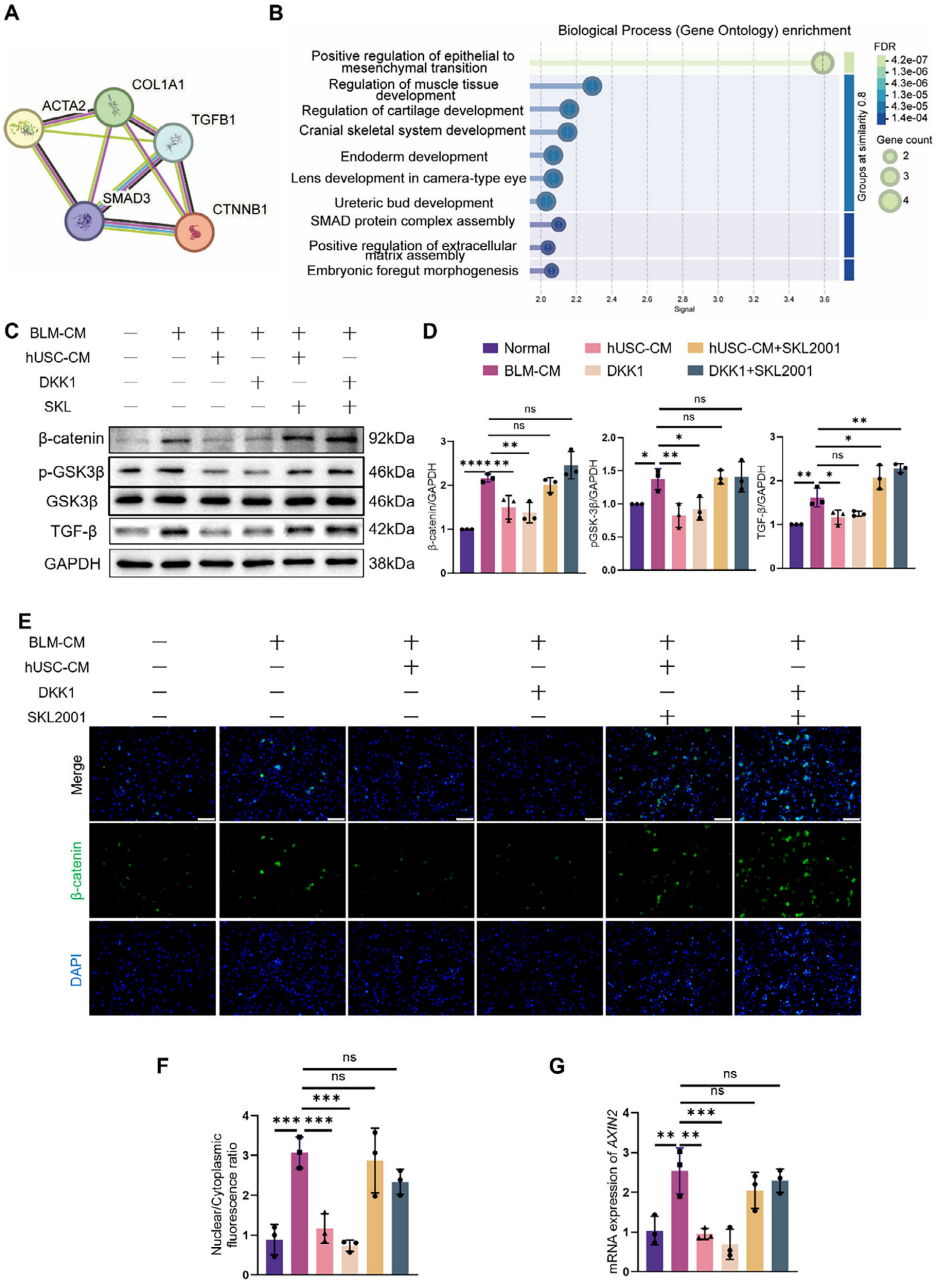
DKK1 attenuated BLM‐induced MMT by inhibiting Wnt/β‐catenin signaling pathway. (A) Protein‐protein interaction (PPI) network of key proteins involved in MMT, fibrosis, and WNT signaling. (B) Gene Ontology (GO) enrichment analysis of fibrosis‐related biological processes. The horizontal bar plot displays enriched terms, with the x‐axis representing the enrichment score. (C, D) Representative western blot images (C) and quantitative analysis (D) of TGF‐β and Wnt/β‐catenin‐related protein expression. (E) Representative immunofluorescence images of β‐catenin in THP‐1 macrophages. (F) The nuclear/cytoplasmic fluorescence ratio was quantified by measuring the mean fluorescence intensity in the nucleus and cytoplasm of each cell. (G) The relative mRNA expression of *AXIN2* were determined by RT‐qPCR in THP‐1, with *GAPDH* as the internal control. Data were represented as mean ± SD. Significance was measured using a one‐way ANOVA. ^*^ *P* < 0.05, ^**^ *P* < 0.01, ^***^ *P* < 0.001, ^****^ *P*<0.0001, n = 3.

We further analyzed the protein‐protein interaction (PPI) network of MMT‐related proteins (α‐SMA, Collagen I), fibrosis‐related proteins (TGF‐β, SMAD3), and Wnt/β‐catenin signaling pathway‐related proteins (β‐catenin) using the STRING database. Potential interactions among β‐catenin, transforming growth factor‐β (TGF‐β), collagen type I (COL1A1), and α‐smooth muscle actin (α‐SMA) were identified (Figure 8A). In addition, Gene Ontology (GO) enrichment analysis revealed that these proteins were highly associated with positively regulating extracellular matrix assembly and epithelial‐mesenchymal transition (Figure 8B). Notably, although epithelial‐mesenchymal transition (EMT) was the most significantly enriched GO term, this might not be simply interpreted as just classical EMT in the pulmonary fibrosis progression. It likely reflects a shared profibrotic mesenchymal‐transition program, as no dedicated GO term is currently available for macrophage‐to‐myofibroblast transition (MMT). To further elucidate the mechanism by which hUSCs inhibit MMT via DKK1, Wnt/β‐catenin agonist SKL2001 was used to restore the inhibited Wnt signaling. The results showed that SKL2001, by stabilizing β‐catenin expression and promoting β‐catenin nuclear translocation, reversed the inhibitory effects of hUSC‐CM or DKK1 on the Wnt/β‐catenin signaling pathway and aggravated MMT (Figure 8C–F). Moreover, the expression of *AXIN2*, a canonical downstream target of Wnt/β‐catenin signaling, was significantly increased in THP‐1 macrophages treated with supernatants from BLM‐injured alveolar epithelial cells, whereas hUSC‐CM or DKK1 markedly suppressed the elevation of *AXIN2*. Notably, SKL2001 restored *AXIN2* expression (Figure 8G). These results indicated that hUSC‐derived DKK1 is involved in the suppression of Wnt/β‐catenin‐associated signaling, monocyte recruitment, and MMT, which might contribute to the anti‐fibrotic effects of hUSCs.

## 3 Discussion

The BLM‐induced pulmonary fibrosis model represents one of the most well‐established and widely utilized preclinical tools in the field [28], although it is not an etiological factor of idiopathic pulmonary fibrosis (IPF) [29, 30]. While the outcomes may differ between the experimental model and the human disease [31], they share core pathological features including inflammatory cell infiltration, fibroblast activation, and excessive extracellular matrix deposition [2]. Thus, the findings derived from the BLM‐PF model should contribute to the common pathophysiological pathways in pulmonary fibrosis and offer valuable experimental support for developing therapeutic strategies for IPF and other fibrotic lung diseases.

This study highlights the protective role of human urine‐derived stem cells (hUSCs) against pulmonary fibrosis via multifaceted effects, especially through paracrine mechanism to suppress MMT. MMT is defined as macrophage‐to‐myofibroblast transition and serves as a key link between inflammatory responses and fibrotic progression [7]. In the early stages of lung injury, macrophages primarily contribute to tissue repair [32]. However, under conditions of sustained injury, a subset of macrophages undergo MMT, directly transforming into myofibroblasts with a pro‐fibrotic phenotype. Through analysis of tissue samples from healthy individuals and patients with IPF, along with validation in a mouse model of fibrosis, we demonstrated that pulmonary fibrosis was closely correlated with macrophage‐to‐myofibroblast transition (MMT), evidenced by consistent co‐expression of macrophage markers (CD68 or F4/80) and α‐smooth muscle actin (α‐SMA, a hallmark protein of myofibroblasts) in pulmonary fibrotic tissues. Notably, these MMT‐derived myofibroblasts not only specifically localize to inflammatory core regions but also continuously secrete transforming growth factor‐beta (TGF‐β), which establishes a self‐amplifying positive feedback loop to drive the ongoing progression of fibrosis. This finding provides a crucial mechanistic explanation for why pulmonary fibrosis tends to progress relentlessly once initiated. Collectively, our data demonstrated that MMT represents an important source of myofibroblasts in pulmonary fibrosis, suggesting that targeting the MMT process or blocking its downstream signaling pathways might offer a novel therapeutic strategy for inhibiting the progressive nature of fibrosis.

Classically, myofibroblasts involved in the progression of pulmonary fibrosis are known to originate from multiple sources, including well‐established epithelial‐mesenchymal transition (EMT) [33] and endothelial‐mesenchymal transition (EndMT) [34]. MMT has been discovered recently as one of the essential mechanisms in fibrosis‐related diseases, including pulmonary fibrosis [35]. EMT classically refers to the differentiation of epithelial cells into mesenchymal cells; thus, MMT and EMT are biologically distinct and arise from different cellular origins. However, they share several profibrotic signaling pathways, including TGF‐β/SMAD and Wnt/β‐catenin, as well as downstream mesenchymal and myofibroblast‐associated gene programs [35]. Because the GO database does not include a dedicated term for macrophage‐to‐myofibroblast transition currently, MMT‐related signatures may be annotated to the nearest available biological process terms, including EMT‐related categories. Therefore, EMT enrichment in our GO analysis might reflect a shared fibrogenic mesenchymal‐transition program rather than classical EMT itself.

In addition, we also demonstrated that the dysregulated clearance function of macrophages was responsible for the irreversibility of pulmonary fibrosis. We observed that the medium derived from A549 cells in the presence of BLM initiated MMT, whereas the supernatants derived from MMT further aggravated the senescence of alveolar epithelial cells (A549 cells), indicating that the effects from dysregulated macrophages were more pronounced than the direct action of bleomycin in alveolar epithelial damage and revealing a reciprocal detrimental manner between alveolar epithelial cells and macrophages. Our results might suggest that macrophage phagocytic function might be dysregulated under a sustained pathological microenvironment, shifting from the normal “clearance of apoptotic cells to promote repair” to the “excessive phagocytosis” of injured epithelial cells. Such aberrant clearance behavior fails to restore tissue homeostasis and may instead exacerbate the disruption of the epithelial barrier, which may act in synergy with the aforementioned mechanism of MMT and drive the malignant progression of fibrosis.

Our findings demonstrated that hUSCs‐based therapy possessed a multi‐layered and synergistic protective effect in modulating the progression of pulmonary fibrosis, which was primarily achieved through the following three core pathways: first, hUSCs significantly suppressed the apoptosis of alveolar epithelial cells induced by BLM, thereby mitigating primary lung tissue damage at the “injury initiation” stage and preventing the triggering of fibrosis; second, hUSCs reduced macrophage recruitment to the injury site by directly inhibiting monocyte chemotaxis and indirectly alleviating epithelial damage, thereby limiting the potential cellular source for subsequent macrophage‐to‐myofibroblast transition (MMT), and lastly and most critically, hUSCs might intervene the process of MMT to block the central link between inflammation and pulmonary fibrosis via DKK1‐associated suppression of Wnt/β‐catenin signalling and reducing TGF‐β production in macrophages. In addition, hUSC‐derived DKK1 might directly bind to its plasma membrane receptor LRP5/6 to prevent Wnt‐Catenin signalling as previously reported [27], and SKL2001, an activator of Wnt‐Catenin signalling, effectively reversed DKK1‐mediated anti‐MMT effects. Certainly, these actions of hUSCs will help to prevent fibroblast activation and aberrant extracellular matrix deposition. Notably, the therapeutic effect of hUSCs is systemic and irreplaceable: it does not target an isolated step but covers the entire pathological cascade from “injury initiation—inflammatory recruitment—fibrotic progression”. In particular, targeting MMT, a key driver of pulmonary fibrosis, distinguishes stem cell‐based therapy mechanistically from existing clinical drugs such as nintedanib and pirfenidone, which primarily alleviate symptoms rather than address the core mechanism, which will provide a novel approach for achieving fundamental intervention of pulmonary fibrosis.

Despite the promising anti‐fibrotic effects observed in this study, several translational challenges should be considered before hUSC‐based therapy can be advanced clinically. Although the serum‐free culture system used here may reduce concerns related to animal‐derived components, large‐scale production for human application would still require GMP‐compliant manufacturing, rigorous quality control, batch consistency, and validated potency assays [36]. In addition, the long‐term safety of intravenous hUSCs delivery remains to be established. Intravascular cell administration may be influenced by pulmonary first‐pass retention and hemocompatibility‐related risks, including coagulation activation and thromboinflammatory reactions [37]. Since we have demonstrated that the therapeutic role of hUSCs in PF is at least partially mediated through paracrine mechanisms involving DKK1 and its modulation of Wnt/β‐catenin‐associated fibrotic responses in MMT, this might suggest a potential application of hUSC‐CM or exosomes in the treatment of PF via alternative delivery such as intratracheal administration. Therefore, future studies should further evaluate large‐scale manufacturing feasibility, biodistribution, repeated‐dose safety, and long‐term outcomes of hUSCs in IPF treatment.

In addition, some limitations should be acknowledged in the present study. First, beyond the effects of hUSCs on macrophages and epithelial cells, they may also modulate other cell types, such as vascular endothelial cells and lymphocytes in the fibrotic lung tissues. Therefore, the synergistic regulatory mechanisms among these diverse cell types may be worth systematically exploring. Second, A549 cells, which have been widely used as alveolar epithelial‐like cells, might not fully recapitulate the biological behavior of primary alveolar type II cells in fibrotic lungs. Primary alveolar epithelial cells or more physiologically relevant human organoid models might be needed for further validation in PF modeling. Third, the direct evidence underlying MMT would be further advantaged by myeloid lineage‐tracing experiments via establishing macrophage‐to‐myofibroblast fate conversion in PF modeling in vivo. Lastly, it might be worth investigating clinically to validate the involvement of Wnt/β‐catenin pathway in regulating pro‐fibrotic MMT and the potential application of hUSCs in IPF.

In conclusion, our data demonstrated that hUSCs protect against pulmonary fibrosis by protecting alveolar epithelial cells from apoptosis and senescence, suppressing the recruitment of monocytes and macrophage‐myofibroblast transition (MMT). In addition, we have provided strong evidence to reveal that macrophage‐myofibroblast transition (MMT) might be a key source of pulmonary fibroblasts in human and mouse pulmonary fibrosis. Mechanically, we demonstrated that hUSCs might mitigate pulmonary fibrosis by suppressing monocyte recruitment and MMT via blocking the Wnt/β‐catenin signaling pathway from extracellular DKK1.

## 4 Experimental Section

### 4.1 Isolation and Culture of hUSCs

Human urine‐derived stem cells (hUSCs) were isolated and cultured in serum‐free medium as previously described [38] with modifications. Briefly, midstream urine (100 mL) was collected from each of 4–6 healthy male donors. Each sample was centrifuged at 400 × g for 10 min at room temperature (RT). The pellet was washed in phosphate‐buffered saline (PBS) three times and resuspended in 1 mL of serum‐free hUSC medium (SF medium)containing 60% MCDB 153 medium (Sigma, USA), 30% MEM α medium (Gibco, USA), 4% human platelet lysate (SEXTON, USA), 1× Insulin‐Transferrin‐Selenium‐Ethanolamine (ITS‐X, Gibco), 1% Non‐Essential Amino Acids (NEAA, Gibco), 1% penicillin/streptomycin (Gibco), 2 × 10^−^^9^ mol/L 3,3′,5‐Triiodo‐L‐thyronine, hydrocortisone (both from Sigma), and 10 ng/mL epidermal growth factor (EGF, Peprotech, USA). The cells were seeded into 12‐well plates and maintained in an incubator at 37°C with 5% CO_2_, and the medium was changed completely every three days. Distinct cobblestone‐like hUSCs colonies typically appeared around 7–10 days and were passaged onto a new 12‐well plate, which was designated as passage 0 (P0). When the cells reached 80% confluence, they were passaged and sequentially scaled up for expansion from a 6‐well plate to 10 cm dishes. The entire procedure for collecting and culturing was kept in a sterile environment to avoid microbial contamination. The cells were further identified by stem cell‐specific markers such as OCT4, NANOG, and SOX2, and were used at P6‐10 for subsequent experiments.

### 4.2 Preparation of hUSCs‐Conditioned Medium

Human urine‐derived stem cells (hUSCs) at passages 6–10 (P6–10) were seeded into 10 cm culture dishes and cultured at 37°C with 5% CO_2_. When hUSCs reached 80%–90% confluence in a 10 cm culturing plate, the cells were changed into basal medium (66% MDCB153 medium, 33% α‐MEM medium, and 1 X ITS) at 10 mL/plate and cultured for an additional 48 h. Then, the culture media were collected and centrifuged at 1500 rpm for 10 min to remove cell debris. The supernatants were harvested as hUSC‐conditioned medium (hUSC‐CM) and transferred to a Millipore ultrafiltration tube (Millipore, UFC900324) and centrifuged at 5000 × g, 60 min at 4°C to 10 fold (10×) concentrated media. Finally, the concentrated 10× hUSC‐CM was stored at −80°C for subsequent usage at 1×.

### 4.3 Animal and Bleomycin‐Induced Pulmonary Fibrosis Model In Vivo

All animal procedures were approved by the Animal Ethics Committee of Nanchang University and conducted in accordance with the Laboratory Animal Guideline for ethical review of animal welfare in China. Male C57BL/6 mice (7–8 weeks old, 22–24 g) were purchased from Jiangsu Jicui Yaokang Biotechnology Co., Ltd and randomly assigned to three groups (n = 10, per group) as the control group (normal saline), the BLM group (modeling), and the BLM + hUSCs group (treatment). Pulmonary fibrosis was induced by a single intratracheal instillation of bleomycin (BLM, Nippon Kayaku Co., Tokyo, Japan) at 3 mg/kg in normal saline under isoflurane anesthesia [23]. Control mice received an equal volume of sterile normal saline. Mice in the BLM + hUSCs group (treatment group) received two intravenous injections of hUSCs at 1 × 10^6^ cells per mouse in 100 µL PBS (passages 6–10) via tail vein on day 7th and 14th post‐BLM administration, respectively. On day 21, lung structural images were acquired by Quantum GX2 Micro‐CT (PerkinElmer, Inc., Waltham, MA, USA). Images were acquired with an intrinsic retrospective two‐phase respiratory gating technique with the following parameters: 70 KV, 100 µA over a total angle of 360° for a total scan time of 4 min. Lung volumes were estimated based on 3D‐reconstituted images.

### 4.4 H&E, Masson's Trichrome, and Sirius Red Staining

Lung tissues were harvested, washed twice with PBS, and fixed in 4% PFA at RT for 24 h, then dehydrated through a graded ethanol series, embedded in paraffin, and sectioned at 4 µm thickness. Histopathological staining was performed on tissue slides after dewaxing and rehydration with commercial kits according to the manufacturers' instructions, such as hematoxylin and eosin (H&E; Solarbio, G1120), Masson's trichrome (Solarbio, G1340), and Sirius Red (Sbjbio, BP‐DL027). The images were captured under a microscope imaging system (EVOS FL Auto, Thermo Fisher Scientific).

### 4.5 Tissue Immunofluorescence Staining

For immunofluorescence staining, paraffin‐embedded tissue sections were dewaxed, rehydrated, and subjected to antigen retrieval using a citrate‐based buffer (pH 6.0). After permeabilization with 0.3% Triton X‐100 for 10 min, sections were blocked with 5% goat serum at room temperature for 1 h., and then incubated with primary antibodies at 1:500 dilution overnight at 4°C, followed by incubation with appropriate fluorescently labeled secondary antibodies at room temperature for 1 h in the dark. Cell nuclei were counterstained with 4′,6‐diamidino‐2‐phenylindole (DAPI). Finally, the sections were mounted with an anti‐fade mounting medium and imaged using a confocal laser scanning microscope (Zeiss LSM 800). Detailed information on the antibodies used was provided in Table S1.

### 4.6 Reverse Transcription‐Polymerase Chain Reaction (RT‐PCR) and Quantitative Real‐Time Reverse Transcription PCR (RT‐qPCR)

Total RNAs were extracted from hUSCs, lung tissues, or A549 cell line or Thp‐1 cell line using Trizol reagent (Invitrogen Life Technologies, 15596026), and the RNA concentration was measured with a spectrophotometer (Nano Drop 2000, Thermo Fisher). The cDNA was first synthesized using the RevertAid First Strand cDNA Synthesis Kit (Thermo Fisher, K1622). For conventional RT‐PCR, PCR amplification was performed using a thermal cycler (Thermo Hybaid, USA), and the PCR products were separated on 2% agarose gels containing 0.5 µg/mL ethidium bromide and visualized under UV light. The primers used for RT‐PCR are listed in Table S4. For RT‐qPCR, the reactions were performed using Hieff qPCR SYBR Green Master Mix (Yeasen, 11202ES08) on an ABI ViiA7 Real‐Time PCR System. Relative mRNA expression was calculated using the 2−ΔΔCt method and normalized to GAPDH. The primers used for RT‐qPCR are listed in Table S5.

### 4.7 Flow Cytometry Analysis of Cytotoxicity, Proliferation, and Cell Cycle

Human alveolar type II epithelial cells (A549) [39] were placed at 6 × 10^5^ cells/well in a 6‐well plate in three different groups: control, BLM modeling (BLM 80 µg/mL), and BLM+hUSC‐CM intervention (BLM 80 µg/mL + hUSC‐CM) and cultured for 48 h, and single resuspended A549 cells were prepared in PBS for subsequent flow cytometry analysis. According to the manufacturer's protocols, corresponding detection kits such as Cell Cycle Analysis Kit (K2263, APExBIO) and Annexin V‐FITC/PI Apoptosis Detection Kit (40302ES20, Yeasen) were used, respectively. To measure cytotoxicity, the cells were incubated in 500 µL binding buffer with Annexin V‐FITC and phosphotidylserine (PI) from the Apoptosis Detection Kit at room temperature in the dark for 15 min and analyzed by flow cytometry (Cytoflex, Beckman Coulter) within 1 h. For cell cycle measurement, single resuspended cells were fixed with 70% ethanol at 4°C overnight, then incubated in working solutions containing PI from the Cell Cycle Analysis Kit in the dark at room temperature for 30 min, followed by flow cytometry.

### 4.8 Live–Dead Cell Assay

Cell viability was assessed with a Live/Dead Cell Staining Kit (Beyotime, C2015S). Briefly, a staining working solution was prepared by mixing PBS with the provided PI and Calcein‐AM stock solutions at a volume ratio of 1000:1:1. After incubating at 37°C for 30 min in the dark, the cells were observed under a fluorescent microscope (Olympus IX83).

### 4.9 β‐Galactosidase Staining

Cell samples in 6‐well plates were washed twice with PBS at room temperature. Senescence‐associated β‐galactosidase (SA‐β‐gal) activity was detected using a commercial staining kit (Solarbio, G1580) according to the manufacturer's protocol. Briefly, cells were fixed with 1 mL of fixative solution per well for 15 min at room temperature. After the cells were washed with PBS three times, the staining working solution was added, and the cells were incubated at 37°C overnight (12–24 h). The cellular senescence was judged by the accumulated SA β‐gal staining.

### 4.10 Western Blotting Analysis

Protein lysates were prepared from lung tissues, A549 cells (human type II alveolar epithelial cell line), and THP‐1 cells (human monocytic cell line) with RIPA buffer supplemented with protease and phosphatase inhibitors. Lysates were centrifuged at 12 000 × rpm for 15 min at 4°C, and protein concentration was quantified by BCA Protein Assay Kit (Thermo Fisher Scientific, Cat. No. 23227). Equal amounts of protein were denatured in loading buffer, separated by 12% SDS‐PAGE, and transferred onto PVDF membranes. After blocking with 5% non‐fat dry milk or 3% BSA in TBST for 1 h at room temperature, the membranes were incubated with primary antibodies diluted in TBST containing 1% BSA overnight at 4°C. Following three washes with TBST, the membranes were incubated with horseradish peroxidase (HRP)‐conjugated secondary antibodies for 1 h at room temperature. Protein bands of interest were visualized using enhanced chemiluminescence (ECL) substrate and imaged with a chemiluminescence imaging system (Tanon 4600, Shanghai). Band intensities were quantified using ImageJ software (National Institutes of Health, USA). Antibody details were provided in Table S1.

### 4.11 Single‐Nucleus RNA‐Seq Data Standardization and Downstream Analysis

A single‐nucleus transcriptome dataset was retrieved from the GEO database (GSE286182). The R package Seurat (version 3.1.0) was used to cluster the cells in the merged matrix. Cells with <200 detected transcripts or >25% mitochondrial gene expression were first filtered out as low‐quality cells. The ‘FindAllMarkers’ function was employed to analyze the differentially expressed genes (DEGs) between normal and diseased states. DEGs were functionally annotated using the DAVID platform, with Gene Ontology (GO) and Kyoto Encyclopedia of Genes and Genomes (KEGG) pathways selected for enrichment analysis.

### 4.12 Cell Communication Analysis

A CellChat object was constructed from the standardized single‐cell data. Subsequently, the ligand‐receptor database was imported, overexpressed genes were identified, and ligand‐receptor relationships were established to infer the cell communication network.

### 4.13 Pseudotime Analysis

To construct the cell differentiation trajectory, we converted the annotated Seurat object into a Monocle3 object. Principal component analysis (PCA) was performed for dimensionality reduction (the top 50 PCs were selected), followed by UMAP dimensionality reduction and cell clustering. Based on the feature of high expressions of “*TGFB1, ACTA2, COL1A1, COL3A1, CD163*”, monocytes were set as the root node of the trajectory, and the cell trajectory graph was learned. Finally, the pseudotime value of each cell was calculated, and genes significantly altered during the differentiation process (q‐value < 0.01) were identified via graph‐based trajectory dependence testing (graph test).

### 4.14 ATII Epithelial Cells and Macrophages Co‐Culture System

A549 cells were seeded in a 24‐well transwell plate (Corning, CLS3422) at 1‐1.5 × 10^5^ cells/well and divided into three groups as normal control group, BLM model group, and hUSC‐CM intervention group, in which they were pre‐incubated with BLM 80 µg/mL for 48 h. THP‐1 macrophages, which were pre‐seeded in 8 µm pore size transwell inserts at 2 × 10^5^ cells/insert and differentiated by 100 ng/mL PMA (P8139, Sigma–Aldrich) by PMA for 24 h, and then the inserts were placed into the respective wells. A549 cells and THP‐1 macrophages were co‐cultured in 1640 medium plus 1% FBS for an additional 48 h to allow macrophages to migrate through the membranes, which were collected and stained with crystal violet and observed under a light microscope (Leica DMI1).

### 4.15 Statistical Analysis

The results were presented as means ± standard deviations (SD). The unpaired t‐test was used for analysis between two groups. One‐way analysis of variance (ANOVA) was used to compare data among three or more groups, as indicated. Differences with a p‐value of < 0.05 were considered statistically significant. The software used for data analysis was Prims 9.5.

## Author Contributions

H.B.X., K.Y.D. and Z.H.Z. conceived and designed the study. Z.H.Z., M.H., D.W.G., and N.L., Y.Q.Z. performed experiments in vitro. Z.H.Z., H.C.G., and Q.M.H. performed in vivo experiments. G.L.G. analyzed the snRNA‐seq data. Z.H.Z. wrote the manuscript draft. H.B.X and K.Y.D. revised the manuscript.

## Conflicts of Interest

The authors declare no conflict of interest.

## Supporting information




**Supporting File 1**: advs76150‐sup‐0001‐SuppMat.docx.

**Supporting File 2**: advs76150‐sup‐0001‐Video1.avi.

## Data Availability

The data that support the findings of this study are available from the corresponding author upon reasonable request.
