# Supplementary material for: Human Urine Stem Cells Alleviate Pulmonary Fibrosis via Inhibiting Macrophage‐Myofibroblast Transition
Source: Adv Sci (Weinh). 2026 Jun 16:e76150. Online ahead of print. doi: 10.1002/advs.76150 (PMC13336853; doi:10.1002/advs.76150)
Supplement: Supplementary file 1 — Supporting File 1: advs76150‐sup‐0001‐SuppMat.docx. [file ADVS-9999-e76150-s001.docx]

**Supporting Information:**


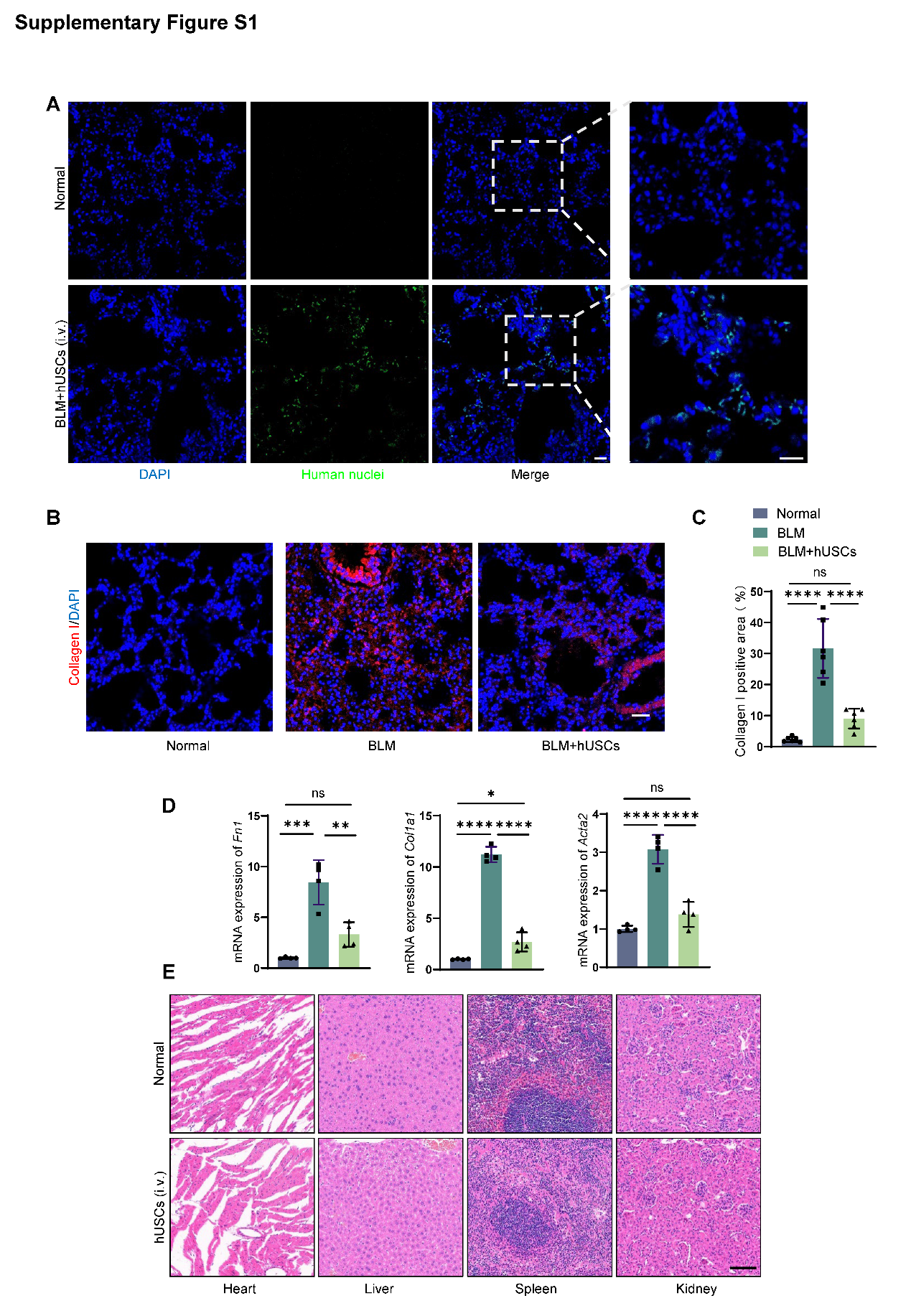


Figure **S1. Assessment of pulmonary fibrosis in mouse lung tissues. (A)** Representative images of human cell nuclei in mouse lung tissues with immunofluorescent detection of MAB1281 antibody, Bars =20μm. **(B)** Representative immunofluorescence images of Collagen I in mouse lung tissues. **(C)** Quantification of Collagen I fluorescence intensity from B. **(D)** The relative mRNA expression of *Fn1, Col1a1* and *Acta2* was determined by RT‑qPCR in mouse lung tissues, with *Gapdh* as the internal control. **(E)** H&E staining of heart, liver, spleen, and kidney sections to detect histopathological alterations, Bar=100μm.Data were represented as mean ± SD. Significance was measured using one‑way ANOVA. *P < 0.05, **P < 0.01, ***P < 0.001, ****P<0.0001. n=4-6.


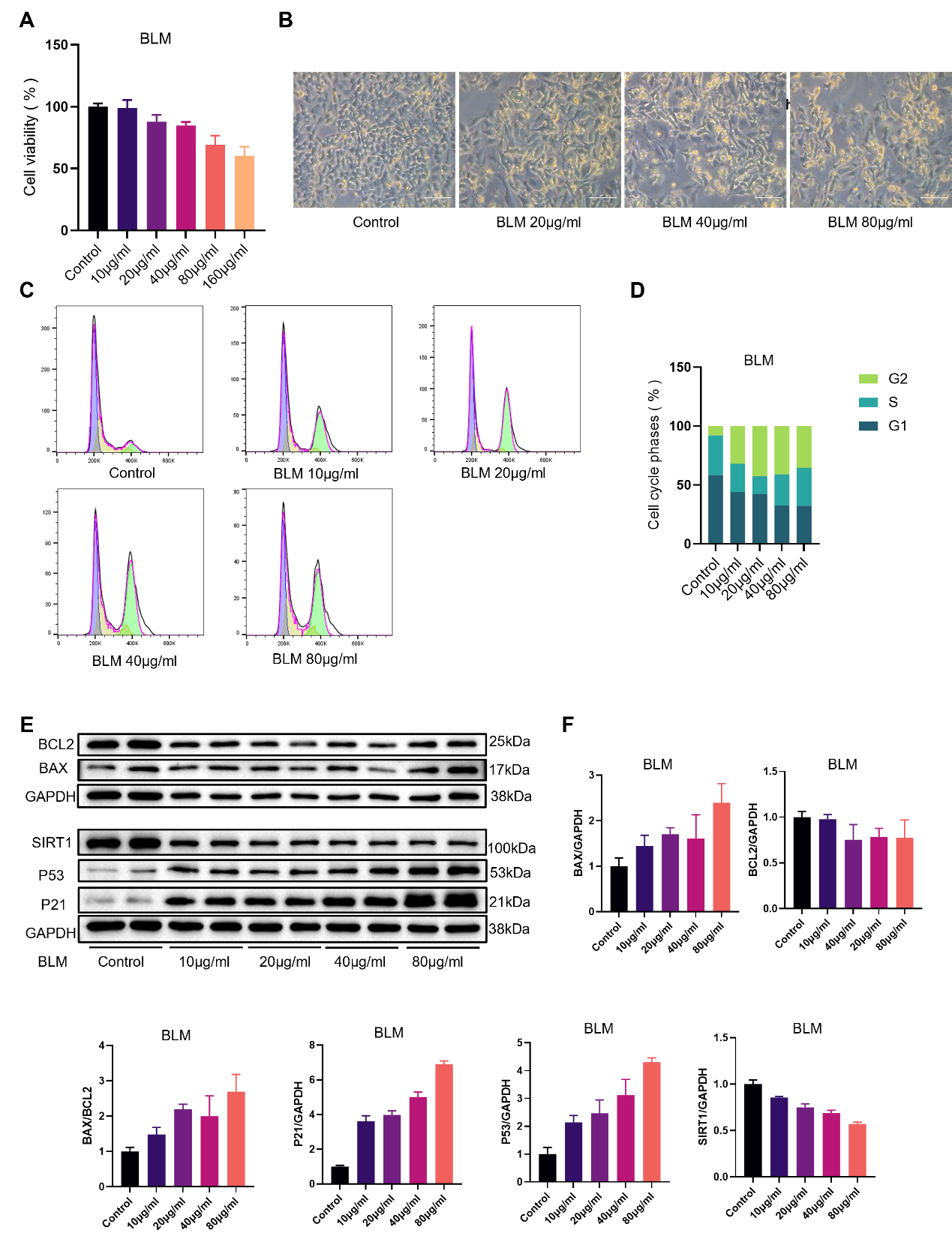


Figure **S2. Reduced cell viability was observed in dose-dependent manner with induced damage and senescence in A549 cell by BLM treatment.** A549 cells were treated with BLM at indicated concentrations (0–160 μg/mL) for 48 h. **(A)** Cell viability measured by CCK-8 assay. **(B)** Bright-field microscopy images showing morphological changes. **(C)** Representative flow cytometry plots of cell cycle distribution. **(D)** Quantification of percentage of cells in each cell cycle phase. **(E)** an**d (F)** Representative western blot bands and statistical analysis of senescence and apoptosis related proteins. Data were represented as mean ± SD, n=3-4.


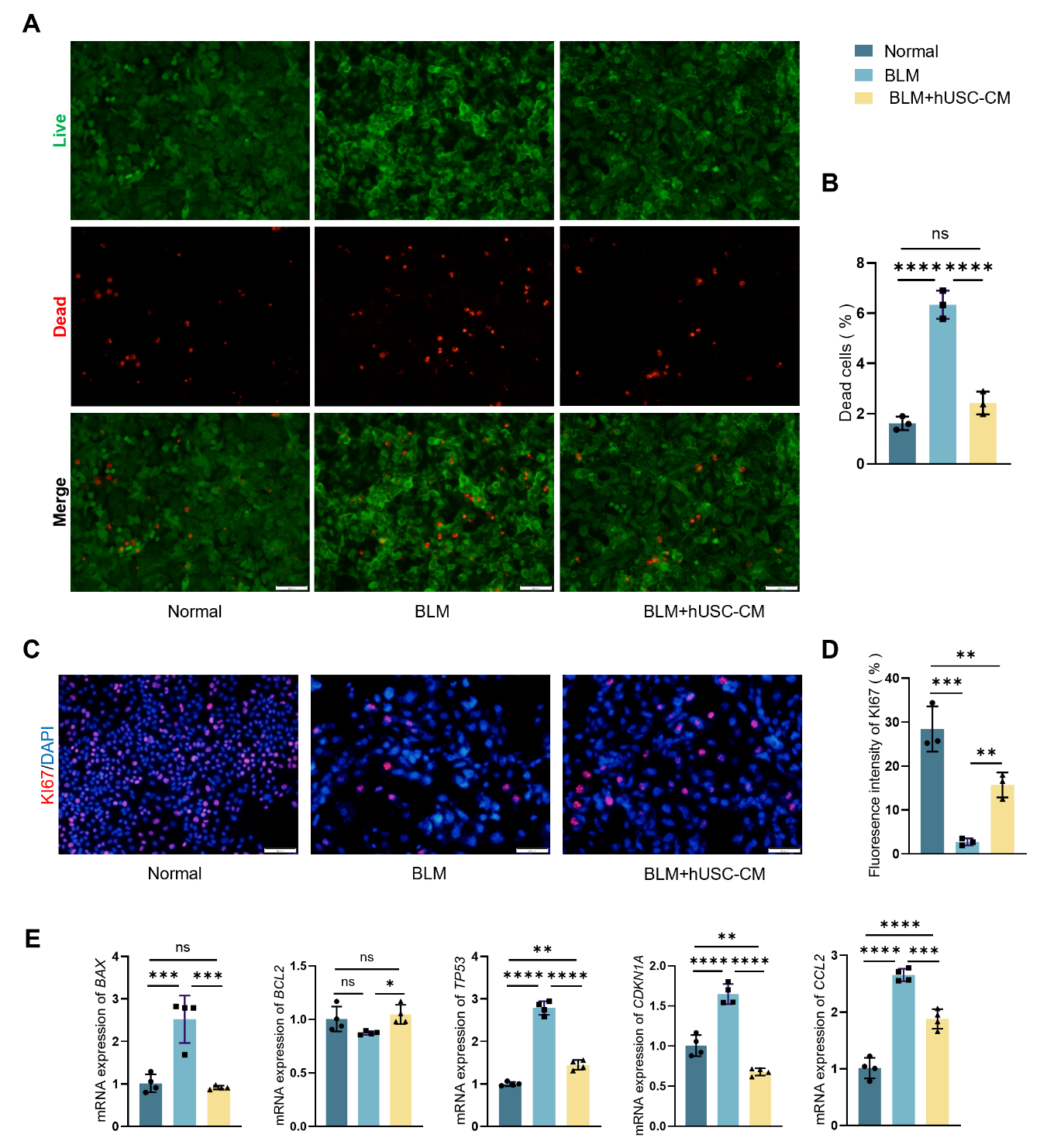


Figure S3. **Effects of hUSC-CM on BLM-induced cell death and proliferation in A549 cells.** A549 cells were treated with BLM (80 μg/ml) with or without hUSC-CM for 48 h. **(A)** Representative live/dead cell staining images (live cells in green; dead cells in red). **(B)** Quantification of dead cell percentage from A. **(C)** Representative immunofluorescence images of Ki67 (proliferation marker). **(D)** Quantification of Ki67-positive cells from C. **(E)** The mRNA expressions of *BAX, BCL2, TP53, CDKN1A* and *CCL2* were determined by RT-qPCR analysis in A549 cell, with *GAPDH* as the internal control. Data were represented as mean ± SD. Significance was measured using one‑way ANOVA. *P < 0.05, **P < 0.01, ***P < 0.001, ****P<0.0001. n=3-4


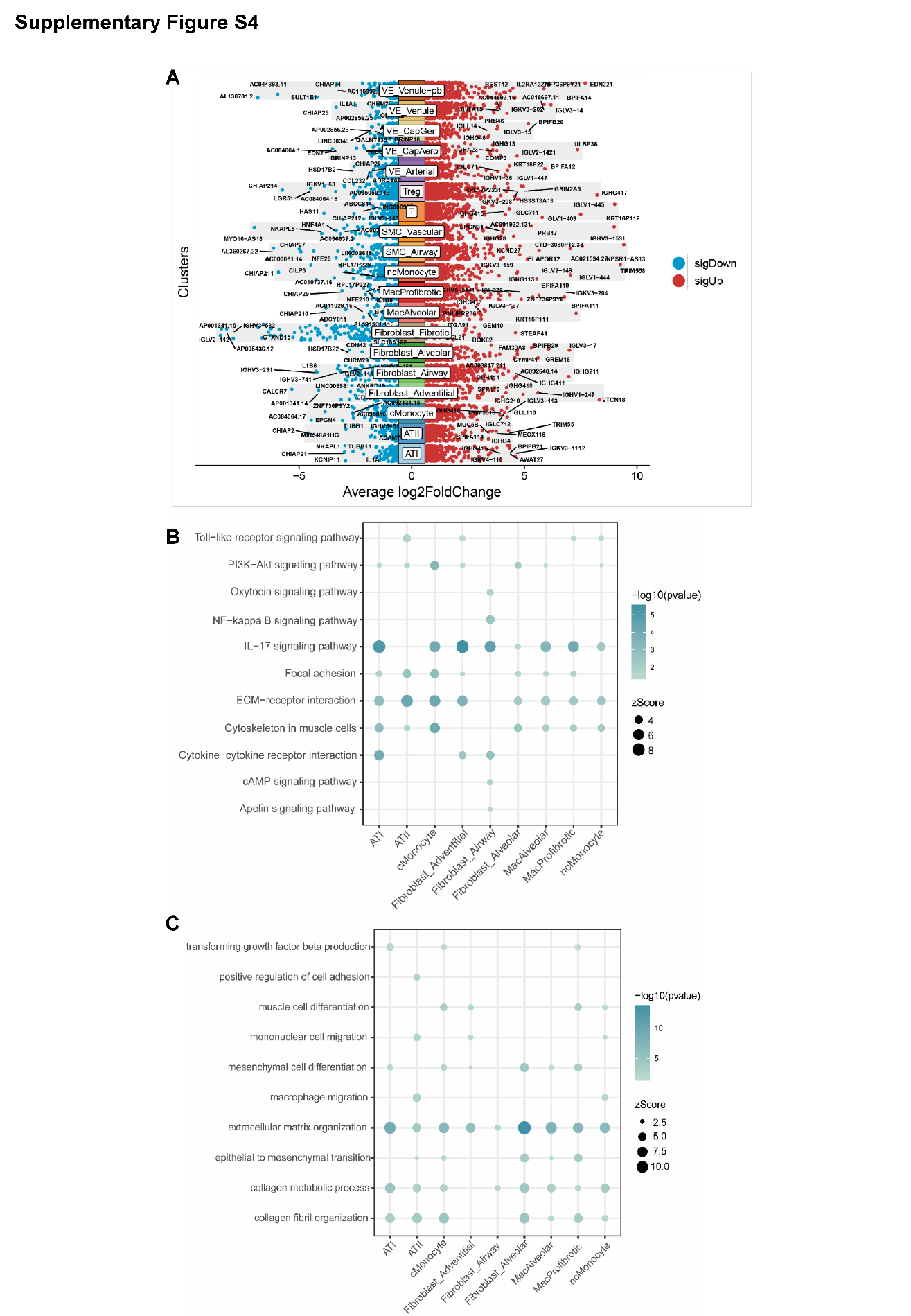


Figure **S4. Bioinformatic analysis of lung tissues from IPF patients and healthy donors. (A)** Bubble plot of differential gene expressions across distinct subpopulation of cells in lung tissues from IPF patients and healthy donors. **(B)** GO enrichment analysis for biological process in differentially expressed genes in lung tissues. **(C)** KEGG analysis for the differentially expressed genes revealed in fibrotic lung tissues.


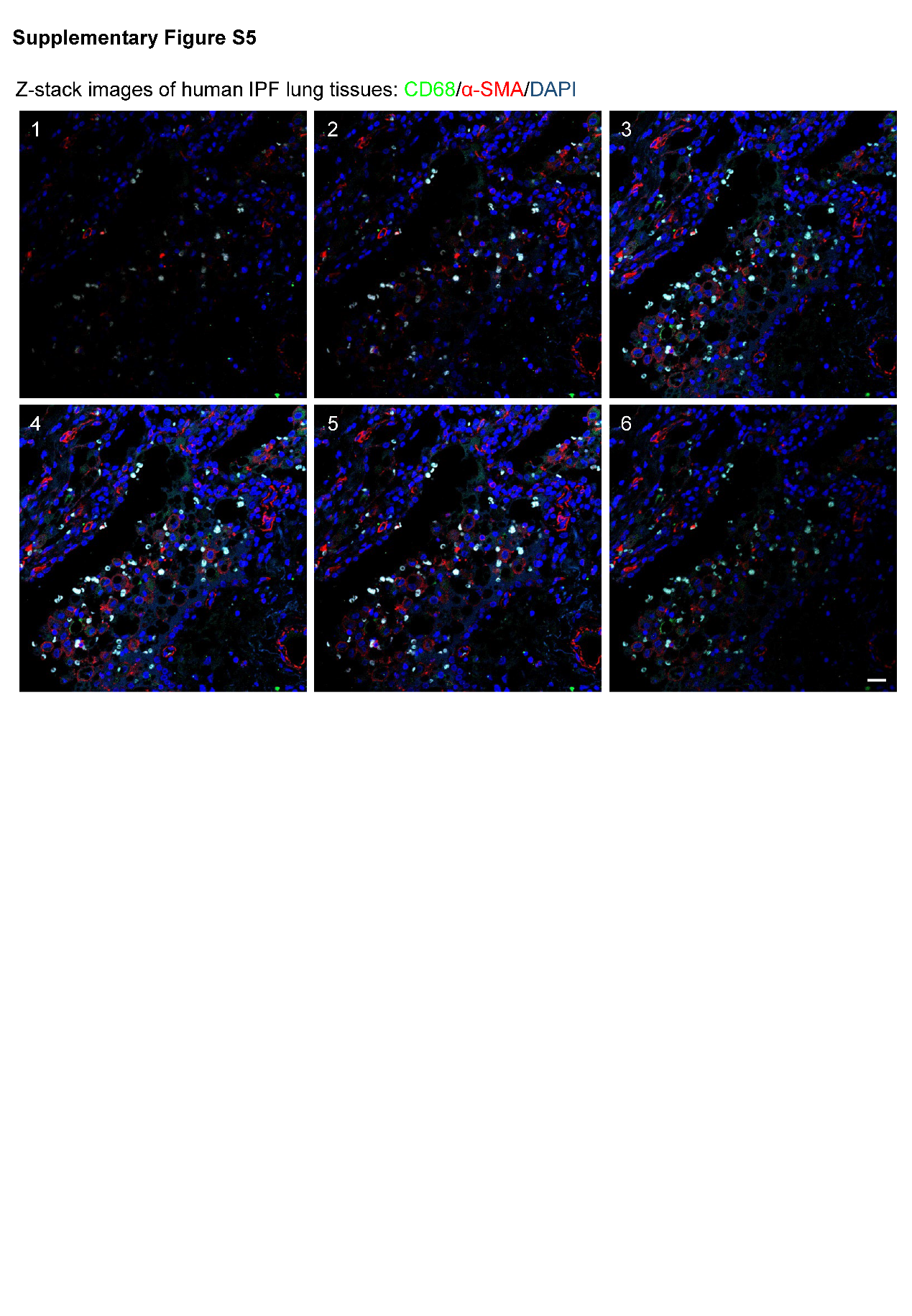


Figure S5. MMT-like cells with CD68^+^/α-SMA^+^ in human IPF lung tissues. Immunofluorescent detection of CD68^+^ (green) and α-SMA^+^ (red) were performed in human IPF lung tissues and the representative Z-stack images captured by Zeiss LSM 800 confocal microscopy were presented with serial number. Bar=20 μm.


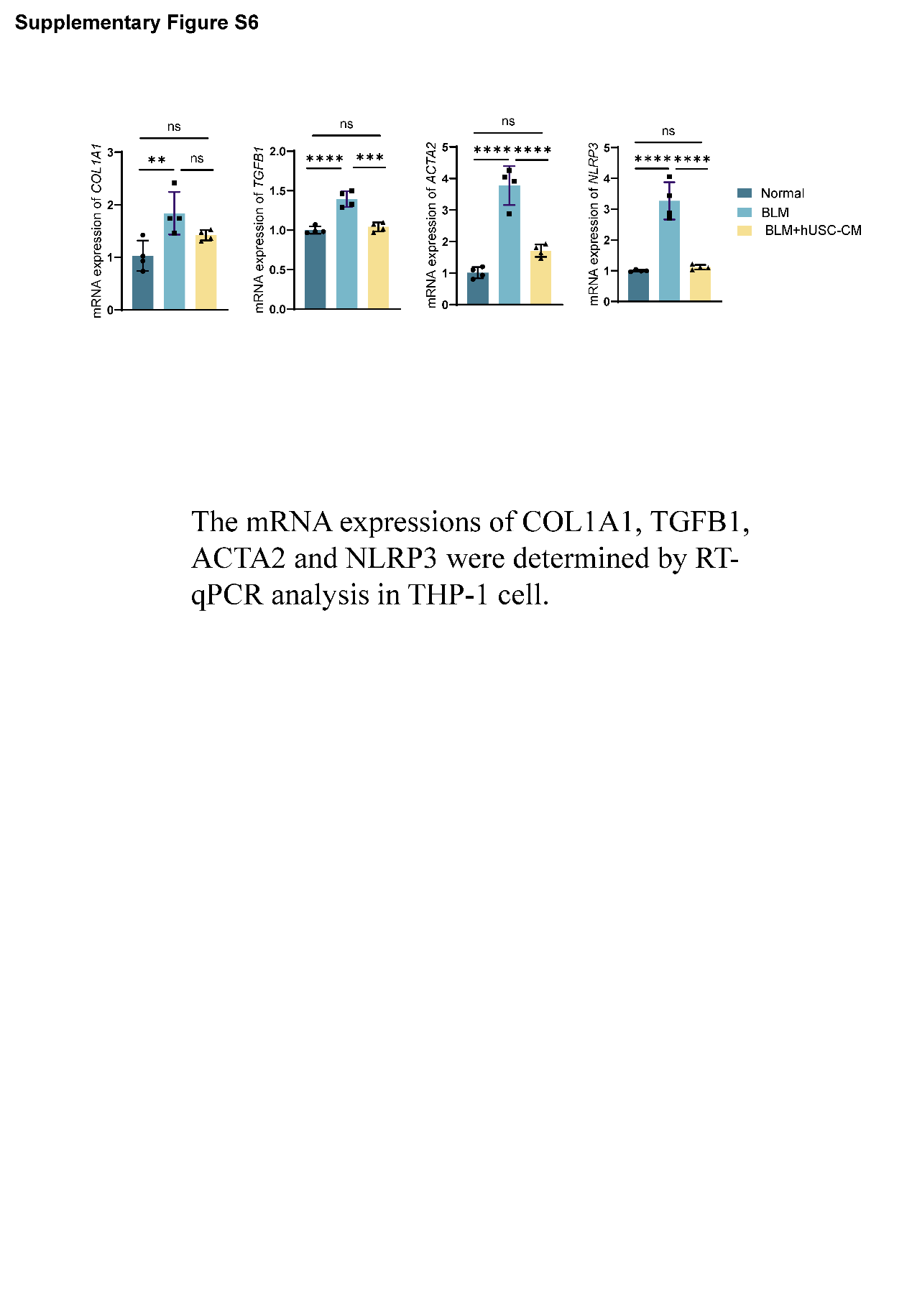


Figure **S6.** The mRNA expressions of *COL1A1, TGFB1, ACTA2* and *NLRP3* were determined by RT-qPCR analysis in THP-1 cell, with *GAPDH* as the internal control. Data were represented as mean ± SD. Significance was measured using a one‑way ANOVA. *P < 0.05, **P < 0.01, ***P < 0.001, ****P<0. 0001, n=4.


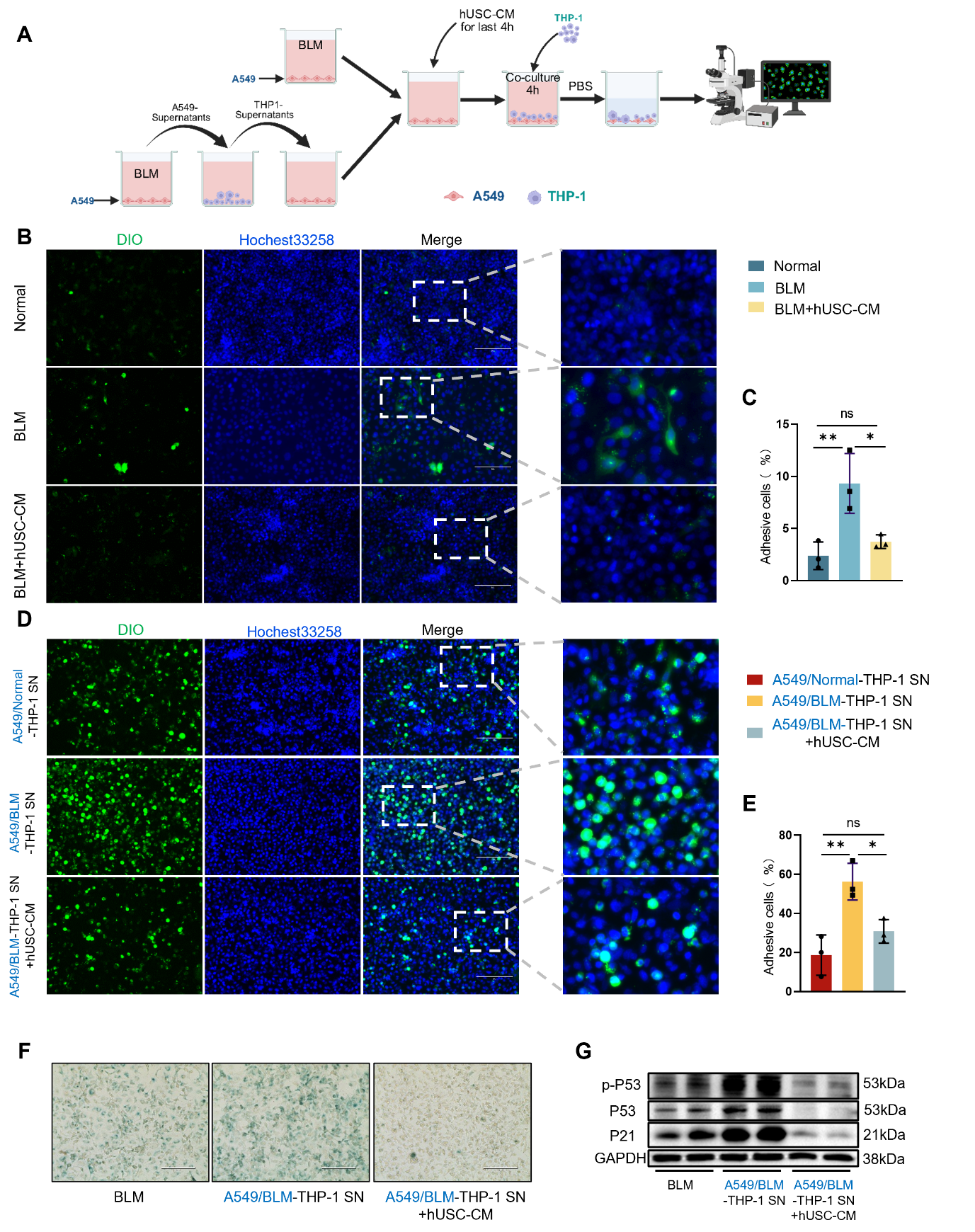


Figure **S7. Effects of hUSC-CM on macrophage recruiment and A549 cell senescence. (A)** Schematic illustration of THP-1/A549 co-culture system. **(B) and (D)** Representative images of macrophage adhesion assay. THP-1 macrophages (green, DiO stain) and A549 cells (blue, Hoechst 33258 stain) are shown. **(C)** and **(E)** Quantification of the rate of macrophage adhesion from panels B and D, respectively. **(F)** Senescence-associated β-galactosidase (SA-β-gal) staining in A549 cells. **(G)** Representative western blot bands of senescence-related proteins in A549 cells. Data were represented as mean ± SD. Significance was measured using a one‑way ANOVA. *P < 0.05, **P < 0.01, ***P < 0.001, ****P<0. 0001, n=3.


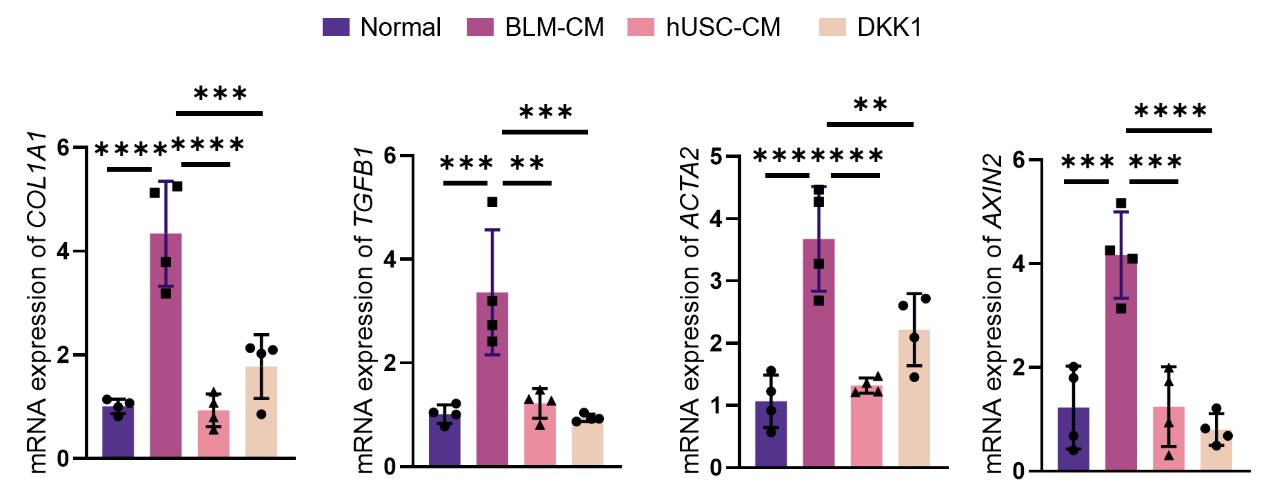


Figure **S8.** The mRNA expressions of *COL1A1*, *TGFB1*, *ACTA2*, and *AXIN2* were determined by RT-qPCR analysis in THP-1 cell, with *GAPDH* as the internal control. Data were represented as mean ± SD. Significance was measured using a one‑way ANOVA. *P < 0.05, **P < 0.01, ***P < 0.001, ****P<0. 0001, n=4.

Table S1:

**Antibody Information**

| **Name** | **Company** | **Cat number** | |
| --- | --- | --- | --- |
| p53 | Abmart | | TA0879 |
| p21 | Abmart | | TD6423 |
| Fibronectin | Abmart | | T59537F |
| Collagen I | Abmart | | P28372-B2F |
| α-SMA | Abmart | | T55295S |
| Bax | Abmart | | T40051F |
| Bcl2 | Abmart | | T40056F |
| NLRP3 | Abmart | | P60622R3F |
| TGF-β | Abmart | | PA2154F |
| β-catenin | Abmart | | M24002F |
| Frizzled 4 | Abmart | | M003762F |
| SP-C | Abmart | | TD6647S |
| β-catenin | Servicebio | | GB150016 |
| CD68 | Proteintech | | 66231-2-Ig |
| F4/80 | Zen-bio | | 263101 |
| Caspase3 | Bosterbio | | PB9188 |
| CD73 | Thermofisher | | 12-0739-42 |
| CD44 | Thermofisher | | 11-0441-82 |
| CD90 | Thermofisher | | A15761 |
| CD105 | Thermofisher | | 12-1057-42 |
| CD34 | Thermofisher | | 11-0349-42 |
| CD45 | Thermofisher | | 11-0451-82 |
| HLA-ABC | Thermofisher | | 12-9983-42 |
| HLA-DR | Thermofisher | | 11-9956-42 |
| OCT4 | Abcam | | Ab200834 |
| SSEA4 | Abcam | | Ab16287 |
| Nanog | Abcam | | Ab109250 |
| Human nuclei | Sigma-aldrich | | MAB1281 |
| Goat Anti-Rabbit IgG (Alexa Fluor® 594) | Huabio | | HA1122 |
| Goat Anti-Mouse IgG (Alexa Fluor® 488) | Huabio | | HA1125 |
|  |  | |  |

Table S2

**Data sources**

| GEO Number | Related article | Type of analysis |
| --- | --- | --- |
| GSE286162 | Alveolar epithelial cell plasticity and injury memory in human pulmonary fibrosis | snRNA-seq analysis |
|  |  |  |

Table S3

**Reagent Information**

| **Name** | **Company** | | **Cat number** |
| --- | --- | --- | --- |
| Bleomycin | Nippon Kayaku Co Ltd |  | |
| CCK8 | Wanleibio | WLA074b | |
| Hydroxyproline Assay Kit | Njjcbio | A030-2-1 | |
| Penicillin-streptomycin | Solarbio | P1400 | |
| Fetal Bovine Serum | Gibico | 12484028 | |
| α-MEM | Gibico | 12571063 | |
| RPMI 1640 | Gibico | C11875500BT | |
| IGF | Peprotech | 100-11R3 | |
| EGF | Peprotech | AF-100-15 | |
| bFGF | Peprotech | 100-18B | |
| Phorbol 12-myristate 13-acetate (PMA) | MCE | HY-18739 | |
| Y27632 | MCE | HY-10071 | |
| Hochest3322 | Beyotime | C1011 | |
| DKK-1 | MCE | HY-P72968 | |
| SLK2001 | MCE | HY-101085 | |
| Cell Cycle Assay Kit | Apexbio | K2263 | |
| TUNEL Apoptosis Detection Kit | Yeason | 40308ES50 | |
| Annexin V-FITC/PI Apoptosis Detection Kit | Yeason | 40302ES20 | |
| Super ECL Detction Reagent | Yeason | 36208ES60 | |
| BCA Protein Assay Kit | Thermo Fischer | 23225 | |
| SA-β-Gal Stain Kit | Solarbio | G1508 | |
| MCDB153 | Sigma-aldrich | M7403 | |
|  |  |  | |
|  |  |  | |
|  |  |  | |
|  |  |  | |
|  |  |  | |
|  |  |  | |
|  |  |  | |
|  |  |  | |
|  |  |  | |
|  |  |  | |
|  |  |  | |
|  |  |  | |
|  |  |  | |

Table S4.

**The primer sequences for RT-PCR.**

| **Genes** | **Sequences (5’ to 3’)** |
| --- | --- |
| h-*OCT4*-F | CGCTGGCTTATAGAAGGTGCT |
| h-*OCT4*-R | TGGCATGCATACACACAAACA |
| h-*SOX2*-F | AACCAGCGCATGGACAGTTA |
| h-*SOX2*-R | GACTTGACCACCGAACCCAT |
| h-*NANOG*-F | CAATGGTGTGACGCAGGGAT |
| h- *NANOG* -R | TGCACCAGGTCTGAGTGTTC |
| h- *CD73*-F | TGAAGTTGTGGGAATCGTTGGA |
| h- *CD73*-R | TGGATTCCATTGTTGCGTTCA |
| h-*CD44*-F | ACTGTACACCCCATCCCAGA |
| h-*CD44*-R | GGCTTGGTGTTGTCCTTCCT |
| h-*CD90*-F | AGGGAGGAAGAGCAGACCTT |
| h-*CD90*-R | AGAGGGTAGAAGAGCCAGGG |
| h-*CD105*-F | TCCTCCCAAGGACACTTGTA |
| h-*CD105*-R | CGCCTCATTGCTGATCATAC |
| h-*GAPDH*-F | CCACCCATGGCAAATTCCATGGCA |
| h-*GAPDH*-R | TCTAGACGGCAGGTCAGGTCCACC |

Table S5.

**The primer sequences for RT-qPCR.**

| **Genes** | **Sequences (5’ to 3’)** |
| --- | --- |
| h-*CTNNB1*-F | CTGAGGAGCAGCTTCAGTCC |
| h-*CTNNB1*-R | CCATCAAATCAGCTTGAGTAGCC |
| h-*AXIN2*-F | TAACCCCTCAGAGCGATGGA |
| h-*AXIN2*-R | AGTTCCTCTCAGCAATCGGC |
| h-*ACTA2*-F | GGGGTCAGCACTTCGCAT |
| h-*ACTA2*-R | GCTTCACAGGATTCCCGTCT |
| h-*COL1A1*-F | AGTGGTTTGGATGGTGCCAA |
| h-*COL1A1*-R | GCACCATCATTTCCACGAGC |
| h-*TGFB1*-F | TACCTGAACCCGTGTTGCTC |
| h-*TGFB1*-R | CCGGTAGTGAACCCGTTGAT |
| h-*GAPDH*-F | AATGGGCAGCCGTTAGGAAA |
| h-*GAPDH*-R | GCGCCCAATACGACCAAATC |
| h-*NLRP3*-F | CTGGCATCTGGGGAAACCT |
| h-*NLRP3*-R | AGCCCTTCTGGGGAGGATAG |
| m-*GAPDH*-F | AGGTCGGTGTGAACGGATTTG |
| m-*GAPDH*-R | TGTAGACCATGTAGTTGAGGTCA |
| m-*trp53*-F | ATTCAGGCCCTCATCCTCCT |
| m-*trp53*-R | CCATGGCAGTCATCCAGTCT |
| m-*Bax*-F | GATCAGCTCGGGCACTTTAG |
| m-*Bax*-R | TTGCTGATGGCAACTTCAAC |
| m-*Bcl2*-F | CTCAGGCTGGAAGGAGAAGAT |
| m-*Bcl2*-R | AAGCTGTCACAGAGGGGCTAC |
| m-*Col1a1*-F | CTGGCGGTTCAGGTCCAAT |
| m-*Col1a1*-R | TTCCAGGCAATCCACGAGC |
